# Association of Chemoradiotherapy Regimens and Survival Among Patients With Nasopharyngeal Carcinoma: A Systematic Review and Meta-analysis

**DOI:** 10.1001/jamanetworkopen.2019.13619

**Published:** 2019-10-18

**Authors:** Bin Zhang, Min Min Li, Wen Hui Chen, Jian Fu Zhao, Wei Qi Chen, Yu Hao Dong, Xiao Gong, Qiu Ying Chen, Lu Zhang, Xiao Kai Mo, Xiao Ning Luo, Jie Tian, Shui Xing Zhang

**Affiliations:** 1 Department of Radiology, The First Affiliated Hospital of Jinan University, Guangzhou, Guangdong, China; 2 Department of Radiation Oncology, The First Affiliated Hospital of Jinan University, Guangzhou, Guangdong, China; 3 Department of Oncology, The First Affiliated Hospital of Jinan University, Guangzhou, Guangdong, China; 4 Big Data Decision Institute, Jinan University, Guangzhou, Guangdong, China; 5 Department of Catheterization Laboratory, Guangdong Cardiovascular Institute, Guangdong Provincial Key Laboratory of South China Structural Heart Disease, Guangdong Provincial People’s Hospital/Guangdong Academy of Medical Sciences, Guangzhou, Guangdong, China; 6 Department of Epidemiology and Biostatistics, School of Public Health, Guangdong Pharmaceutical University, Guangzhou, Guangdong, China; 7 Department of Head and Neck Cancer, Guangdong Provincial People’s Hospital/Guangdong Academy of Medical Sciences, Guangzhou, Guangdong, China; 8 Guangdong Provincial People’s Hospital/Guangdong Academy of Medical Sciences, Guangzhou, Guangdong, China; 9 Key Laboratory of Molecular Imaging, Chinese Academy of Sciences, Beijing, China

## Abstract

**Importance:**

The role of induction chemotherapy (IC) or adjuvant chemotherapy (AC) in the treatment of locoregionally advanced nasopharyngeal carcinoma (NPC) remains controversial.

**Objectives:**

To update meta-analyses on the association of survival outcomes with IC and AC regimens in patients with locoregionally advanced NPC and assess whether the current evidence is conclusive by a trial sequential analysis (TSA) approach.

**Data Sources:**

PubMed, Embase, and Web of Science were searched for articles published from inception until June 1, 2019.

**Study Selection:**

Randomized clinical trials that assessed the efficacy of radiotherapy with or without chemotherapy among previously untreated patients and patients with nondistant metastatic NPC.

**Data Extraction and Synthesis:**

Data were extracted by 2 investigators from each trial independently and synthesized by the 2 investigators. All trial results were combined and analyzed by a fixed- or random-effects model.

**Main Outcomes and Measures:**

Overall survival (OS), progression-free survival (PFS), distant metastasis–free survival (DMFS), and locoregional recurrence-free survival (LRFS).

**Results:**

A total of 8036 patients (median age, 46.5 years; 5872 [73.1%] male) from 28 randomized clinical trials were included in the analysis. Pooled analyses revealed that concurrent chemoradiotherapy (CCRT) was significantly associated with improved OS, PFS, DMFS, and LRFS compared with radiotherapy across all subgroups. The TSA confirmed the treatment outcomes of CCRT compared with radiotherapy. The additional IC regimen was associated with an improvement in OS (hazard ratio [HR], 0.84; 95% CI, 0.74-0.95), PFS (HR, 0.73; 95% CI, 0.64-0.84), DMFS (HR, 0.67; 95% CI, 0.59-0.78), and LRFS (HR, 0.74; 95% CI, 0.64-0.85). These findings were consistent in subgroup analyses of multicenter trials with sample sizes greater than 250, years of survival rate of 5 or greater, median follow-up longer than 5 years, or low risk of bias. However, the additional AC regimen was not associated with a survival benefit in OS (HR, 0.98; 95% CI, 0.78-1.23), PFS (HR, 0.86; 95% CI, 0.70-1.07), DMFS (HR, 0.84; 95% CI, 0.64-1.10), or LRFS (HR, 0.80, 95% CI, 0.59-1.09). The TSA provided sound evidence on the additional benefit of IC but not AC.

**Conclusions and Relevance:**

These data suggest a significant association of survival outcomes with CCRT in patients with locoregionally advanced NPC. The addition of IC instead of AC could achieve survival benefits. The potential therapeutic gain of AC should be explored in the future.

## Key Points

**Question:**

Is induction chemotherapy or adjuvant chemotherapy associated with additional survival benefit in locoregionally advanced nasopharyngeal carcinoma?

**Findings:**

In a systematic review and meta-analysis of 28 randomized clinical trials of 8036 patients, concurrent chemoradiotherapy was associated with substantial improvement in survival outcomes for patients with locoregionally advanced nasopharyngeal carcinoma. Survival benefit was also associated with the addition of induction chemotherapy but not adjuvant chemotherapy to concurrent chemoradiotherapy.

**Meaning:**

For locoregionally advanced nasopharyngeal carcinoma, concurrent chemoradiotherapy should be recommended as the standard treatment strategy, with the addition of induction chemotherapy but not adjuvant chemotherapy.

## Teaser

This systematic review and meta-analysis assesses whether concurrent chemoradiotherapy is associated with improvement in survival outcomes for patients with locoregionally advanced nasopharyngeal carcinoma.

## Introduction

Nasopharyngeal carcinoma (NPC) is characterized by distinct geographic distribution and is particularly prevalent in East and Southeast Asia.^1^ In endemic areas, more than 70% of patients present with advanced (stage III-IV) disease at the time of diagnosis.^2,3^ Despite advances in diagnosis and multimodality treatment, approximately 30% of high-risk patients experience tumor recurrence, with distant metastasis as the primary source of treatment failure.^4,5^ Radiotherapy remains the primary treatment modality because of the anatomical location and radiosensitivity.^6^ Control of early-stage disease with radiotherapy is usually successful, with 5-year overall survival (OS) of 87% to 96%; however, the outcome of locoregionally advanced disease is unsatisfactory, with 5-year OS of 67% to 77%.^7^ Platinum-based concomitant chemotherapy (CCRT) is now the standard treatment for locoregionally advanced NPC, which can significantly reduce local and distant failure.^8^

Additional cycles of chemotherapy, such as the addition of induction chemotherapy (IC) or adjuvant chemotherapy (AC) to CCRT or radiotherapy, may improve distant control in patients at high risk of distant failure.^9^ Induction chemotherapy offers the advantages of early eradication of micrometastases, tumor downstaging, and good tolerability.^9^ Cisplatin, fluorouracil, and docetaxel is the recommended induction regimen for head and neck cancer because of its superiority over cisplatin and fluorouracil.^10,11,12^ Gemcitabine and cisplatin have been established as the first-line treatment of choice over cisplatin and fluorouracil for patients with recurrent or metastatic disease.^13^ A recent randomized phase 3 trial found that additional gemcitabine and cisplatin IC has excellent efficacy and decreased toxicity.^14^ As for AC, cisplatin and fluorouracil are the recommended regimen for locoregionally advanced NPC. However, approximately 60% of patients could not complete the 3 cycles of AC after CCRT.^15^ Although dozens of randomized clinical trials (RCTs) have been conducted, the results were mixed. Therefore, the additional value of IC or AC is still controversial. The treatment guidelines allow for multiple treatment options. On the basis of the foregoing reasons, we decided to perform a meta-analysis, including recent eligible trials, to mainly explore the role of IC, CCRT, and AC regimens in the treatment of locoregionally advanced NPC and to examine whether and when the current evidence is sufficient and whether additional research by the trial sequential analysis (TSA) approach is needed.

## Methods

This meta-analysis was approved by the First Affiliated Hospital of Jinan University Institutional Review Board. The methods and reporting of this systematic review and meta-analysis followed the Preferred Reporting Items for Systematic Reviews and Meta-analyses (PRISMA) reporting guideline.^16,17^

### Eligibility Criteria

The eligible trials met the following PICOS (participants, interventions, comparisons, outcomes, and study design) criteria. The participants were patients with previously untreated, non–distant metastatic, newly histologically confirmed NPC. The interventions and comparisons consisted of radiotherapy plus chemotherapy compared with radiotherapy or a treatment regimen with 1 chemotherapy time compared with the same treatment strategy with chemotherapy at another time. At least 1 of the following outcomes could be extracted directly from the contents of the article or indirectly by the methods of Tierney et al^18^: time-to-event data, including OS, progression-free survival (PFS), distant metastasis–free survival (DMFS), and locoregional recurrence-free survival (LRFS). Only RCTs were included for analysis. This meta-analysis was limited to human studies published in English. For multiple articles from the same institution, articles that reported on different populations during nonoverlapping intervals or trials by different authors were included. Only the latest update was included if there were 2 or more articles about the same trial in the same patient population.

### Search Strategy and Study Selection

We searched PubMed, Embase, and Web of Science for all eligible RCTs from inception to June 1, 2019. The search strategy is presented in eTable 1 in the Supplement. Two independent investigators (B.Z. and M.M.L.) first screened the titles and abstracts to determine whether the citation met the eligibility criteria. They screened the full text for potentially relevant trials when both agreed that a citation met the eligibility criteria. Disagreements between the investigators were resolved by consensus and, if necessary, consultation with a third investigator (Q.Y.C.).

### Data Collection

The 2 independent investigators (B.Z. and M.M.L.) extracted data from the selected RCTs by standardized collection forms and created tables for the trial characteristics and treatment outcomes. Disagreements between the 2 investigators were resolved by consensus and, if necessary, consultation with a third investigator (Q.Y.C.). In particular, if the hazard ratios (HRs) and 95% CIs were available directly in a trial, then the values were used. If not, extraction of summary statistics from an individual trial was performed according to the methods detailed by Parmar et al.^19^

### Assessment of the Quality of Studies

The 2 independent investigators (B.Z. and M.M.L.) performed risk assessment using the Cochrane Collaboration risk of bias tool.^20^ The selected RCTs were assessed for (1) random sequence generation (selection bias), (2) allocation concealment (selection bias), (3) blinding of participants and personnel (performance bias), (4) blinding of outcome assessment (detection bias), (5) incomplete outcome data (attrition bias), (6) selective reporting (reporting bias), and (7) other sources of bias. Each domain was assessed as of low, unclear, or high risk of bias. The highest risk of bias for any criterion was used to reflect the overall risk of bias. Trials were judged to have low risk of bias when all items were assessed to be low risk, trials were judged to have moderate risk of bias when 1 or more items were assessed to be of unclear risk, and trials were judged to have high risk of bias when 1 or more items were assessed to be of high risk.

### Outcomes of Interest

The primary outcomes were OS, PFS, DMFS, and LRFS. Overall survival was defined as the time from randomization until death from any cause. Progression-free survival was defined as the time from randomization to first progression (locoregional or distant) or death from any cause. Distant metastasis–free survival was defined as the time from randomization to first distant metastasis. Locoregional recurrence-free survival was defined as the time from randomization to locoregional recurrence. If both locoregional failure and distant failure occurred at the same time, patients were considered to have an event for distant failure only. The secondary outcomes were the rates of severe (grade 3-5) toxic effects.

### Statistical Analysis

All statistical analyses were performed by RevMan software, version 5.3.3 (Cochrane Collaboration) and Stata software, version 14.0 (StataCorp), and the fixed- or random-effects model was used for analyses. Dichotomous variables were analyzed by the Mantel-Haenszel method and expressed as HRs with 95% CIs. A 2-tailed *P* < .05 was considered to be statistically significant.

Statistical heterogeneity was assessed by the χ^2^ test and the *I*^2^ test, with χ^2^ *P* < .10 or an *I*^2^ greater than 50% considered substantial.^21^ The possibility of publication bias was assessed by visual estimate of funnel plot and by the Egger test or Begg test when at least 10 trials were pooled.^22^ We conducted prespecified subgroup analyses, which were planned for the following variables: (1) chemotherapy regimens (with or without IC, CCRT, or AC); (2) study center design (single-center or multicenter); (3) publication year (before 2015 vs after 2015); (4) sample size (>250 vs ≤250); (5) period of recruitment (>5 vs ≤5 years); (6) survival rate (≤3 vs ≥5 years); (7) World Health Organization histologic type (including type I or not); (8) tumor stage (including stage II or not); (9) median follow-up (>60 vs ≤60 months); and (10) risk of bias (low bias vs moderate or high bias). A fixed-effects or random-effects model was used to estimate odds ratios (ORs) for the comparison of severe toxic effects between 2 groups.

Cumulative meta-analyses are at risk of producing type I error caused by sparse data and repeated significance testing of accumulating data, whereas the TSA can reduce the risk of type I error and estimate the a priori information size (APIS) needed for achieving a preset power level, drawing benefit boundaries and harm boundaries, and calculating futility.^23,24^ The TSA was conducted to explore whether cumulative data are adequately powered to reach a sound conclusion and whether further studies are needed.^25^ The TSA was performed using Stata software, version 14.0, with the random-effects model. The APIS was calculated and the monitoring boundaries were computed by the O’Brien-Fleming approach.^26^ An optimal information size was considered as a 2-sided 5% risk of a type I error, 20% risk of a type II error (power of 80%), and a priori relative risk reduction of 20%. The mean survival rate and loss to follow-up of patients in the selected studies were calculated for the APIS. Cumulative random-effects meta-analysis with Lan-DeMets bounds was used to calculate TSA-adjusted 95% CIs.

## Results

### Study Selection and Study Characteristics

A total of 28 RCTs^9,14,27,28,29,30,31,32,33,34,35,36,37,38,39,40,41,42,43,44,45,46,47,48,49,50,51,52^ (8036 patients; median age, 46.5 years; 5872 [73.1%] male) were selected for this current meta-analysis. A flowchart of study selection is presented in eFigure 1 in the Supplement. The inclusion criteria and exclusion criteria for selecting trials are presented in eTable 2 in the Supplement.

The characteristics of the included trials are summarized in the Table. Most trials were conducted in endemic areas in East and Southeast Asia, mainly in China (eFigure 2 in the Supplement). A total of 13 comparisons^9,14,27,28,29,30,31,32,33,34,35,36,37^ (4222 patients) investigated IC, including 4 trials^27,28,29,30^ (1209 patients) with the addition of IC to radiotherapy and 9 trials^9,14,31,32,33,34,35,36,37^ (3013 patients) with the addition of IC to CCRT in the treatment group. Four comparisons^38,39,40,41^ (1001 patients) investigated AC, including 2 trials^40,41^ (618 patients) with the addition of CCRT in the groups. Seven comparisons^41,42,43,44,45,46^ (1598 patients, 1 trial with 2 comparisons) investigated CCRT, including 1 trial^41^ (111 patients) with the addition of AC in both groups and 1 trial^46^ (400 patients) with the addition of IC in both groups. Eight comparisons^41,47,48,49,50,51,52^ (1437 patients, 1 trial with 2 comparisons) investigated CCRT plus AC vs radiotherapy. The median follow-up ranged from 30 to 128.4 months. Most trials (17 [61%] of 28) were multicenter trials. Patients from 16 trials (57%) had stage III or IV cancer, and the remaining 12 trials (43%) had patients with stage II cancer. Patients from 16 trials (57%) had a World Health Organization histologic type II or III cancer, and patients from 11 trials (39%) had a World Health Organization histologic type I cancer.

**Table.** Summary of Studies Included in the Meta-analysis

Source	No. of Patients	Study Center Design	Period of Recruitment	Clinical Stage^a^	WHO Type	Median Follow-up, mo	Radiotherapy	Chemotherapy		
								IC	CC	AC
Italy-79,^39^ 1988	229	Multicenter	1979-1983	II-IV^a^	I-III^b^	42, 44^c^	Primary tumor, base of the skull, and involved nodes: 60-70 Gy; negative cervical nodes: 50 Gy	None	None	6 Cycles every 4 wk: vincristine, 1.2 mg/m^2^, day 1, cyclophosphamide, 200 mg/m^2^, days 1-4, and doxorubicin, 40 mg/m^2^, day 1
VUMCA 89/1,^27^ 1996	339	Multicenter	1989-1993	IV	I-III	49	Primary tumor: 65-70 Gy, clinically involved nodes: 65 Gy, remaining cervical and supraclavicular nodal area: 50 Gy	3 Cycles every 3 wk: bleomycin, 15 mg, day 1, 12 mg/m^2^, days 1-5, epirubicin, 70 mg/m^2^, day 1, cisplatinum, 100 mg/m^2^, day 1	None	None
INT-0099,^47^ 1998	147	Multicenter	1989-1995	III, IV	I-III^b^	32.4	Primary tumor: 70 Gy, negative nodes: 50 Gy, positive nodes: 66-70 Gy	None	3 Cycles every 3 wk: cisplatin, 100 mg/m^2^	3 Cycles every 4 wk: cisplatin, 80 mg/m^2^, day 1, and 5-fluorouracil, 1 g/m^2^, days 1-4
AOCOA,^28^ 1998	334	Multicenter	1989-1993	II-IV^a^	II, III	30	Primary tumor: 66-74 Gy, neck: 60-76 Gy	2-3 Cycles every 3 wk: cisplatin, 60 mg/m^2^, day 1, epirubicin, 110 mg/m^2^, day 1	None	None
Guangzhou-93,^30^ 2001	456	Single center	1993-1994	III, IV	I-III	62	Primary tumor: 68-72 Gy, involved areas of the neck: 60-62 Gy, uninvolved areas: 50 Gy	2-3 Cycles every 3 wk: cisplatin, 100 mg/m^2^, day 1, bleomycin, 10 mg/m^2^, days 1-5, and fluorouracil, 800 mg/m^2^, days 1-5	None	None
TCOG-94,^38^ 2002	154	Multicenter	1994-1999	III, IV	I-III	49.5	Primary tumor: 70-72 Gy	None	None	9 Cycles every week: cisplatin, 20 mg/m^2^, fluorouracil, 2200 mg/m^2^, leucovorin, 120 mg/m^2^
Japan-91,^29^ 2002	80	Multicenter	1991-1998	I-IV^a^	I-III	49	Primary tumor: 66-68 Gy, involved lymph nodes: 66-68 Gy, remaining cervical and supraclavicular lymph node areas: 50 Gy	2 Cycles every 3 wk: cisplatin, 80 mg/m^2^, day 1, fluorouracil: 800/m^2^, days 2-5	None	None
Taiwan-93,^44^ 2003	284	Single center	1993-1999	III, IV	I-III	65	Primary tumor and positive nodes: 70-74 Gy, neck: 50-60 Gy	None	2 Cycles every 4 wk: cisplatin, 20 mg/m^2^, days 1-4, and fluorouracil, 400 mg/m^2^, days 1-4	None
QMH-95-01,^41^ 2004	108	Single center	1995-2001	II-IV^a^	I-III	37	Primary tumor: 68 Gy, nodes: 66 Gy, and 10-Gy boost dose for pharyngeal extension and residual nodes	None	Uracil and tegafur in 4:1 molar ratio, 200 mg/d, 7 d/wk	None
QMH-95-02,^41^ 2004	110	Single center	1995-2001	II-IV^a^	I-III	37	Primary tumor: 68 Gy, nodes: 66 Gy, and 10-Gy boost dose were given for pharyngeal extension and residual nodes	None	Uracil and tegafur in 4:1 molar ratio, 200 mg/d 7 d/wk	6 Cycles every 3 wk: alternating cisplatin, 100 mg/m^2^, day 1, and fluorouracil, 1 g/m^2^ daily, days 1-3, and vincristine, 2 mg, bleomycin, 30 mg, and methotrexate, 150 mg/m^2^
QMH-95-03,^41^ 2004	112	Single center	1995-2001	II-IV^a^	I-III	37	Primary tumor: 68 Gy, nodes 66 Gy, and 10-Gy boost dose were given for pharyngeal extension and residual nodes	None	Uracil and tegafur in 4:1 molar ratio, 200 mg/d 7 d/wk	6 Cycles every 3 wk: alternating cisplatin, 100 mg/m^2^, day 1, and fluorouracil 1 g/m^2^ daily, days 1-3, and vincristine, 2 mg, bleomycin, 30 mg, and methotrexate 150 mg/m^2^
QMH-95-04,^41^ 2004	111	Single center	1995-2001	II-IV^a^	I-III	37	Primary tumor 68 Gy, nodes: 66 Gy and 10-Gy boost dose were given for pharyngeal extension and residual nodes	None	Uracil and tegafur in 4:1 molar ratio, 200 mg/d7 d/wk	6 Cycles every 3 wk: alternating cisplatin, 100 mg/m^2^, day 1, and fluorouracil, 1 g/m^2^ per day, days 1-3, vincristine, 2 mg, bleomycin, 30 mg, and methrotrexate, 150 mg/m^2^
PWHQEH-94,^42^ 2005	350	Multicenter	1994-1997	II-IV^a^	I-III^b^	66	Nasopharynx: 66 Gy, parapharyngeal extension: 10- to 20-Gy boost, residual neck nodes and/or residual nasopharyngeal disease: 24-Gy boost (brachytherapy)	None	8 weekly: cisplatin 40 mg/m^2^, day 1	None
SQNP01,^50^ 2005	221	Single center	1997-2003	II-IV	II, III	38.4	Primary tumor: 70 Gy, neck: 60 Gy, positive nodes: 10-Gy boost	None	3 Cycles every 3 wk: cisplatin, 25 mg/m^2^, days 1-4	3 Cycles every 4 wk: cisplatin, 20 mg/m^2^, days 1-4, and fluorouracil 1, g/m^2^, days 1-4
NPC 008,^33^ 2009	65	Single center	2002-2004	III, IV	II, III	51.6	Primary tumor: 66 Gy, residual boost: 7.5 Gy, and parapharyngeal boost: 20 Gy	2 Cycles every 3 wk: docetaxel, 75 mg/m^2^, day 1, and cisplatin, 75 mg/m^2^, day 1	8 Cycles weekly: cisplatin, 40 mg/m^2^	None
Guangzhou 2003,^43^ 2011	230	Multicenter	2003-2007	II, IIIa	II, III	60	Primary tumor: 68-70 Gy, involved neck regions: 60-62 Gy	None	30 mg/m^2^ cisplatin every week	None
NPC-9902-AF,^49^ 2011	96	Multicenter	1999-2004	III, IV	II, III	75.6	Primary tumor: ≥66 Gy, neck: ≥50 Gy, boost: ≤20 Gy when indicated	None	3 Cycles every 3 wk: cisplatin, 100 mg/m^2^	3 Cycles every 4 wk: cisplatin, 80 mg/m^2^, day 1, and fluorouracil, 1 g/m^2^, days 1-4
NPC-9902-CF,^49^ 2011	93	Multicenter	1999-2004	III, IV	II, III	75.6	Primary tumor: ≥66 Gy, neck: ≥50 Gy, boost: ≤20 Gy when indicated	None	3 Cycles every 3 wk: cisplatin, 100 mg/m^2^	3 Cycles every 4 wk: cisplatin, 80 mg/m^2^, day 1, and fluorouracil, 1 g/m^2^, days 1-4
HeCOG,^32^ 2012	141	Single center	2003-2008	II-IV^a^	I-III^b^	55	Primary tumor: 66-70 Gy, involved nodes <3 cm: 60 Gy, nodes ≥3 cm: 70 Gy, and 50 Gy to uninvolved cervical and supraclavicular areas	3 Cycles every 3 wk: epirubicin, 75 mg/m^2^, paclitaxel, 175 mg/m^2^, day 1, and cisplatin, 75 mg/m^2^, day 2	7 Cycles (1 wk for 7 wk): cisplatin, 40 mg/m^2^	None
Guangzhou 2002-2001,^48^ 2013	316	Single center	2002-2005	III, IV	II, III	70	Primary tumor: ≥66 Gy, involved neck: 60-66 Gy, 50 Gy for potential sites	None	3 Cycles every 3 wk: cisplatin, 100 mg/m^2^	3 Cycles every 4 wk: cisplatin, 80 mg/m^2^, day 1, and fluorouracil, 800 mg/m^2^,days 1-5
Guangzhou 2001,^45^ 2013	115	Single center	2001-2003	III, IV	II, III	114	Primary tumor: 70-74 Gy, involved neck: 60-64 Gy, uninvolved neck: 50 Gy	None	6 Cycles weekly: oxaliplatin, 70 mg/m^2^	None
Singapore 2004,^34^ 2015	172	Single center	2004-2012	III, IV	II, III	40.8, 38.4^d^	Primary tumor and pathologic lymph nodes: 70 Gy, the uninvolved neck: 60 Gy	3 Cycles every 3 wk: gemcitabine, 1000 mg/m^2^, carboplatin area under the concentration time curve 2.5, and paclitaxel, 70 mg/m^2^, days 1 and 8	8 Cycles weekly: cisplatin, 40 mg/m^2^	None
Guangzhou 2002,^46^ 2015	400	Single center	2002-2005	II-IV^a^	II, III	133.3	Primary tumor: 66-78 Gy, involved areas of neck: 60-70 Gy	2 Cycles weekly: floxuridine and carboplatin, 750 mg/m^2^, days 1-5	3 Cycles every 3 wk: carboplatin, 750 mg/m^2^	None
Guangzhou 2011,^9^ 2016	480	Multicenter	2011-2013	III, IV	II, III	71.5	Primary tumor: ≥66 Gy, bilateral cervical lymph nodes and potential sites of local infiltration: ≥50 Gy	3 Cycles every 3 wk: docetaxel, 60 mg/m^2^, day 1, cisplatin, 60 mg/m^2^, day 1, and fluorouracil, 600 mg/m^2^, days 1-5	2 or 3 Cycles every 3 wk: cisplatin, 100 mg/m^2^	None
Guangzhou 2008,^31^ 2017	476	Multicenter	2008-2015	III, IV	II, III	50	For 2-dimensional radiotherapy, primary tumor: 64-72 Gy, involved areas of the neck: 60-66 Gy, and uninvolved areas: 48-50 Gy; for IMRT, primary tumor: ≥66 Gy, bilateral cervical lymph nodes and potential sites of local infiltration: ≥50 Gy	2 Cycles every 3 wk: cisplatin, 80 mg/m^2^, day 1, fluorouracil, 800 mg/m^2^, days 1-5	Cisplatin, 80 mg/m^2^, every 3 wk	None
Guangzhou 2006,^40^ 2017	508	Multicenter	2006-2010	III, IV	II, III	68.4	Primary tumor: ≥66 Gy, involved neck: 60-66 Gy, 50 Gy for potential sites	None	7 Cycles weekly of cisplatin, 40 mg/m^2^	3 Cycles every 4 wk: cisplatin, 80 mg/m^2^, day 1, and fluorouracil, 800 mg/m^2^, days 1-5
Guangzhou 2009,^37^ 2017	639	Single center	2009-2012	II-IV^a^	II, III	58.5, 58.2^e^	Primary tumor: 66-72 Gy, involved lymph nodes: 62-70 Gy	2-3 Cycles every 3 wk: cisplatin, 80-100 mg/m^2^, day 1, fluorouracil, 800-1000 mg/m^2^	3 Cycles every 3 wk: cisplatin, 80-100 mg/m^2^, day 1	None
NPC-9903,^51^ 2017	348	Multicenter	1999-2004	III, IV	II, III	128.4	Primary tumor: ≥66 Gy, potential sites of local infiltration and bilateral cervical lymphatics: ≥50 Gy	None	3 Cycles every 3 wk: cisplatin, 100 mg/m^2^	3 Cycles every 4 wk: cisplatin, 80 mg/m^2^, fluorouracil, 1000 mg/m^2^
GORTEC 2006-02,^35^ 2018	81	Multicenter	2009-2012	II-IV^a^	II, III	43.1	Primary tumor: 70 Gy	3 Cycles every 3 wk: docetaxel, 75 mg/m^2^, day 1, cisplatin, 75 mg/m^2^, day 1, and fluorouracil, 750 mg/m^2^, days 1-5	Cisplatin, 40 mg/m^2^, weekly	None
TCOG1303,^36^ 2018	479	Multicenter	2003-2009	IV	I, II	72	Primary tumor: ≥70 Gy, involved neck: 66-70 Gy	3 Cycles every 3 wk: mitomycin, 8 mg/m^2^, epirubicin, 60 mg/m^2^, and cisplatin, 60 mg/m^2^, day 1, fluorouracil, 450 mg/m^2^, and leucovorin, 30 mg/m^2^, day 8	Cisplatin, 30 mg/m^2^, weekly	None
NPC-0502,^52^ 2018	104	Multicenter	2006-2015	IIB-IV^a^	NA	79.2	Primary tumor: ≥66 Gy	None	3 Cycles every 3 wk: cisplatin, 100 mg/m^2^; or 7 cycles weekly of cisplatin, 40 mg/m^2^	6 Cycles every 3 wk: cisplatin, 40 mg/m^2^, gemcitabine, 1000 mg/m^2^, days 1 and 8
Guangzhou 2013,^14^ 2019	480	Multicenter	2013-2016	III, IV	II, III	42.7	Primary tumor: 66-70 Gy, involved cervical lymph nodes: 64-70 Gy	3 Cycles every 3 wk: gemcitabine, 1 g/m^2^, days 1 and 8, cisplatin, 80 mg/m^2^, day 1	3 Cycles every 3 wk: cisplatin, 100 mg/m^2^	None

Abbreviations: AC, adjuvant chemotherapy; AF, accelerated fractionation; AOCOA, Asian-Oceanian Clinical Oncology Association; CC, concurrent chemotherapy; CF, conventional fractionation; GORTEC, Head and Neck Radiation Oncology Group; HeCOG, Hellenic Cooperative Oncology Group; IC, induction chemotherapy; IMRT, intensity-modulated radiotherapy; INT-0099, SWOG (Southwest Oncology Group)–coordinated Intergroup trial, also known as SWOG 8892; NA, not available; NPC, nasopharyngeal carcinoma; PWHQEH, Prince of Wales Hospital, Queen Elizabeth Hospital; QMH, Queen Mary Hospital; SQNP, Singapore Naso-Pharynx; TCOG, Taiwan Cooperative Oncology Group; VUMCA, International Nasopharynx Cancer Study Group; WHO, World Health Organization.

^a^ Significant (>5%) amount of stage I/II disease.

^b^ Significant (>5%) amount of type I disease.

^c^ Median follow-up was 42 months for the control group and 44 months for the experimental group.

^d^ Median follow-up was 40.8 months for experimental group and 38.4 months for control group.

^e^ Median follow-up was 58.5 months for the experimental group and 58.2 months for the control group.

### Risk of Bias of Eligible Studies

Among the 28 selected trials, 17 (61%) were judged as having overall low risk of bias because these trials met all criteria (eFigure 3 and eFigure 4 in the Supplement).

### Primary Clinical End Points and Trial Sequential Analysis

**Figure 1.**
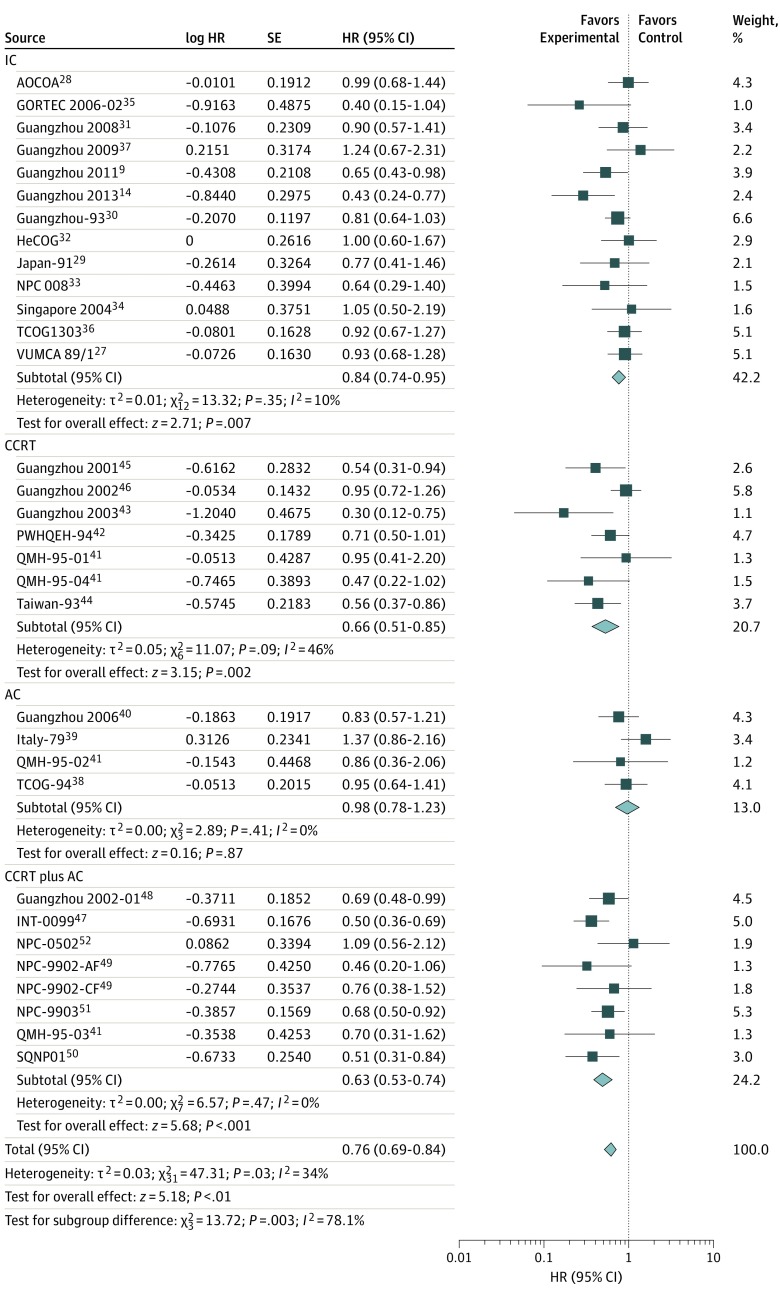
Overall Survival With Hazard Ratios (HRs) by Timing of Chemotherapy The center of each square is the HR for individual trial comparison, with the corresponding horizontal line showing the 95% CIs. The size of the square is proportional to the number of deaths from the trial. The center of the first 4 diamonds is the HR for different timings of chemotherapy, and the extremities are the 95% CIs. The center of the diamond at the bottom represents the overall pooled HR, with the extremities of the diamond showing the 95% CI. AC indicates adjuvant chemotherapy; AF, accelerated fractionation; AOCOA, Asian-Oceanian Clinical Oncology Association; CCRT, concomitant chemoradiotherapy; CF, conventional fractionation; GORTEC, Head and Neck Radiation Oncology Group; HeCOG, Hellenic Cooperative Oncology Group; IC, induction chemotherapy; INT-0099, Southwest Oncology Group (SWOG)–coordinated Intergroup trial (also known as SWOG 8892); NPC, nasopharyngeal carcinoma; PWHQEH, Prince of Wales Hospital, Queen Elizabeth Hospital; QMH, Queen Mary Hospital (2 × 2 design, counted twice in the analysis); SQNP, Singapore Naso-Pharynx; TCOG, Taiwan Cooperative Oncology Group; and VUMCA, International Nasopharynx Cancer Study Group.

**Figure 2.**
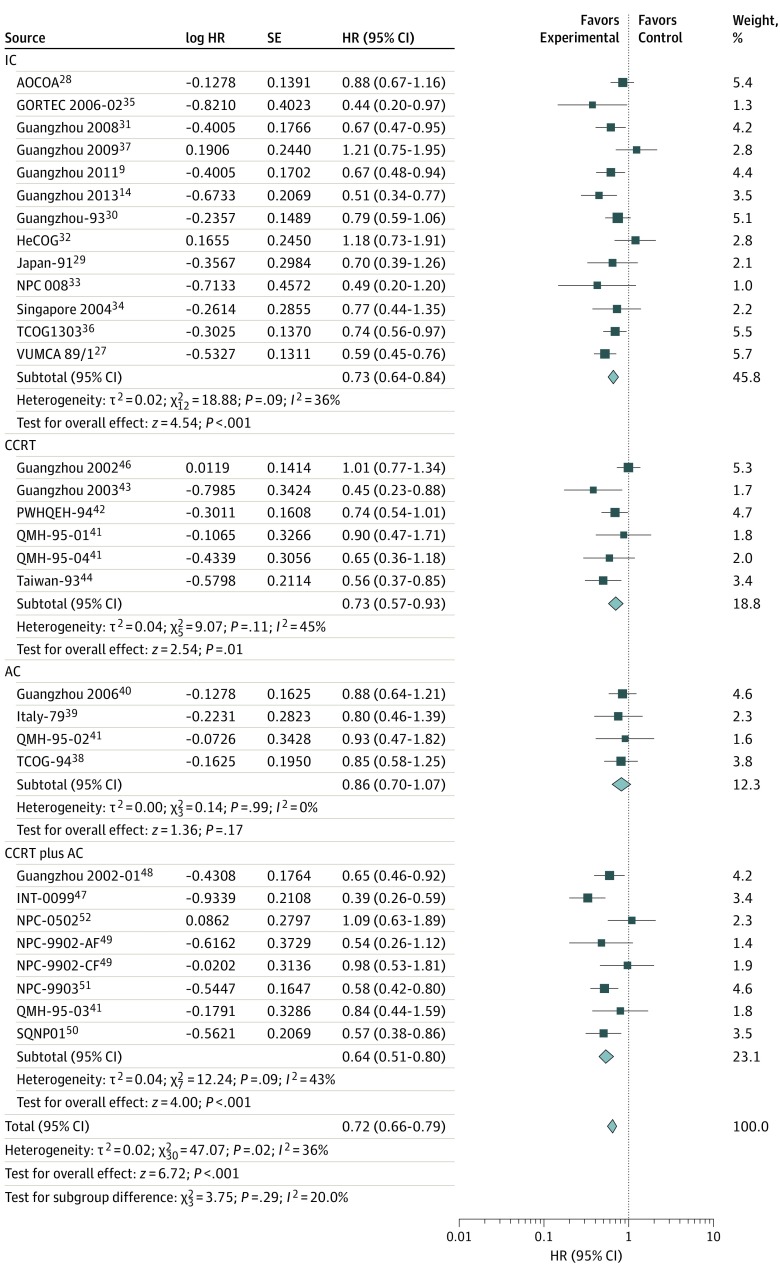
Progression-Free Survival With Hazard Ratios (HRs) by Timing of Chemotherapy The center of each square is the HR for individual trial comparison, with the corresponding horizontal line showing the 95% CI. The size of the square is proportional to the number of relapses or deaths from the trial. The center of the first 4 diamonds is the HR for different timings of chemotherapy, and the extremities are the 95% CIs. The center of the diamond at the bottom represents the overall pooled HR, with the extremities of the diamond showing the 95% CI. AC indicates adjuvant chemotherapy; AF, accelerated fractionation; AOCOA, Asian-Oceanian Clinical Oncology Association; CCRT, concomitant chemoradiotherapy; CF, conventional fractionation; GORTEC, Head and Neck Radiation Oncology Group; HeCOG, Hellenic Cooperative Oncology Group; IC, induction chemotherapy; INT-0099, SWOG (Southwest Oncology Group)–coordinated Intergroup trial (also known as SWOG 8892); NPC, nasopharyngeal carcinoma; PWHQEH, Prince of Wales Hospital, Queen Elizabeth Hospital; QMH, Queen Mary Hospital (2 × 2 design, counted twice in the analysis); SQNP, Singapore Naso-Pharynx; TCOG, Taiwan Cooperative Oncology Group; and VUMCA, International Nasopharynx Cancer Study Group.

**Figure 3.**
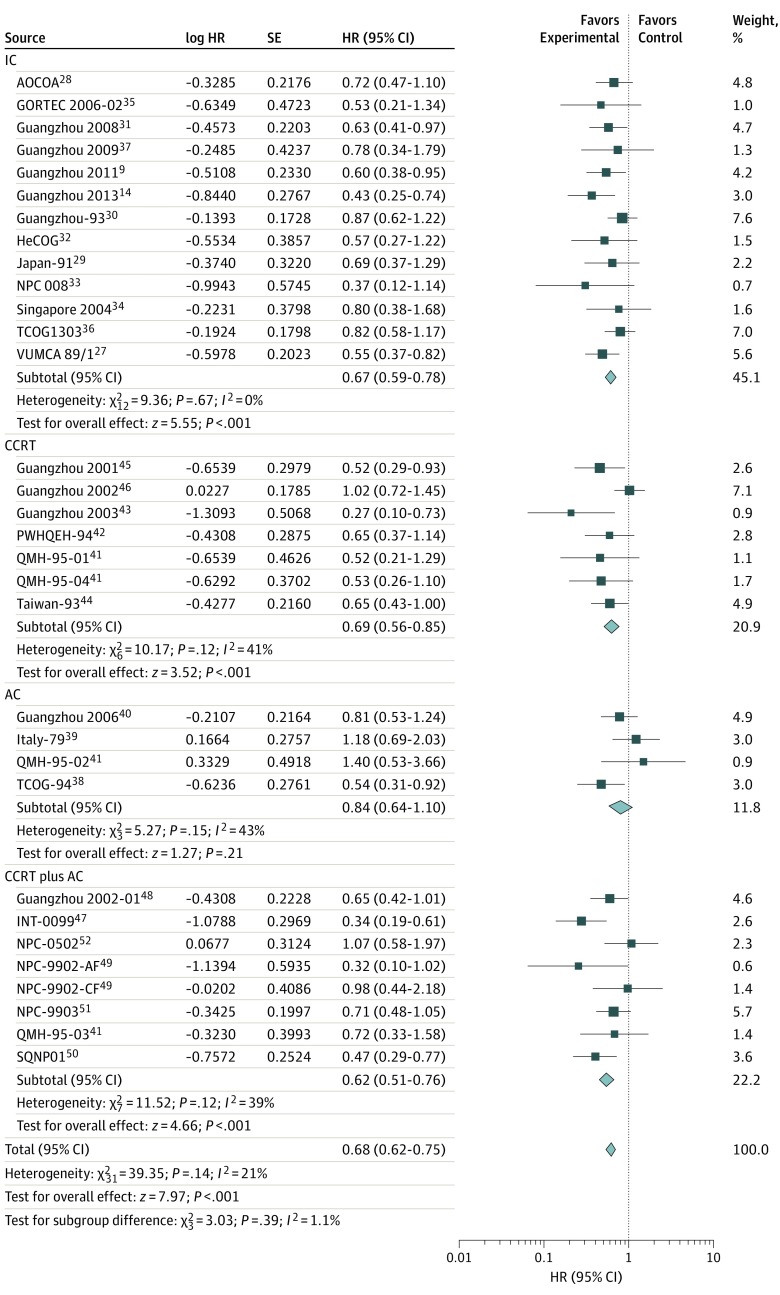
Distant Metastasis–Free Survival With Hazard Ratios (HRs) by Timing of Chemotherapy The center of each square is the HR for individual trial comparison, with the corresponding horizontal line showing the 95% CI. The size of the square is proportional to the number of distance metastasis from the trial. The center of the first 4 diamonds is the HR for different timings of chemotherapy, and the extremities are the 95% CIs. The center of the diamond at the bottom represents the overall pooled HR, with the extremities of the diamond showing the 95% CI. AC indicates adjuvant chemotherapy; AF, accelerated fractionation; AOCOA, Asian-Oceanian Clinical Oncology Association; CCRT, concomitant chemoradiotherapy; CF, conventional fractionation; GORTEC, Head and Neck Radiation Oncology Group; HeCOG, Hellenic Cooperative Oncology Group; IC, induction chemotherapy; INT-0099, SWOG (Southwest Oncology Group)–coordinated Intergroup trial (also known as SWOG 8892); NPC, nasopharyngeal carcinoma; PWHQEH, Prince of Wales Hospital, Queen Elizabeth Hospital; QMH, Queen Mary Hospital (2 × 2 design, counted twice in the analysis); SQNP, Singapore Naso-Pharynx; TCOG, Taiwan Cooperative Oncology Group; and VUMCA, International Nasopharynx Cancer Study Group.

**Figure 4.**
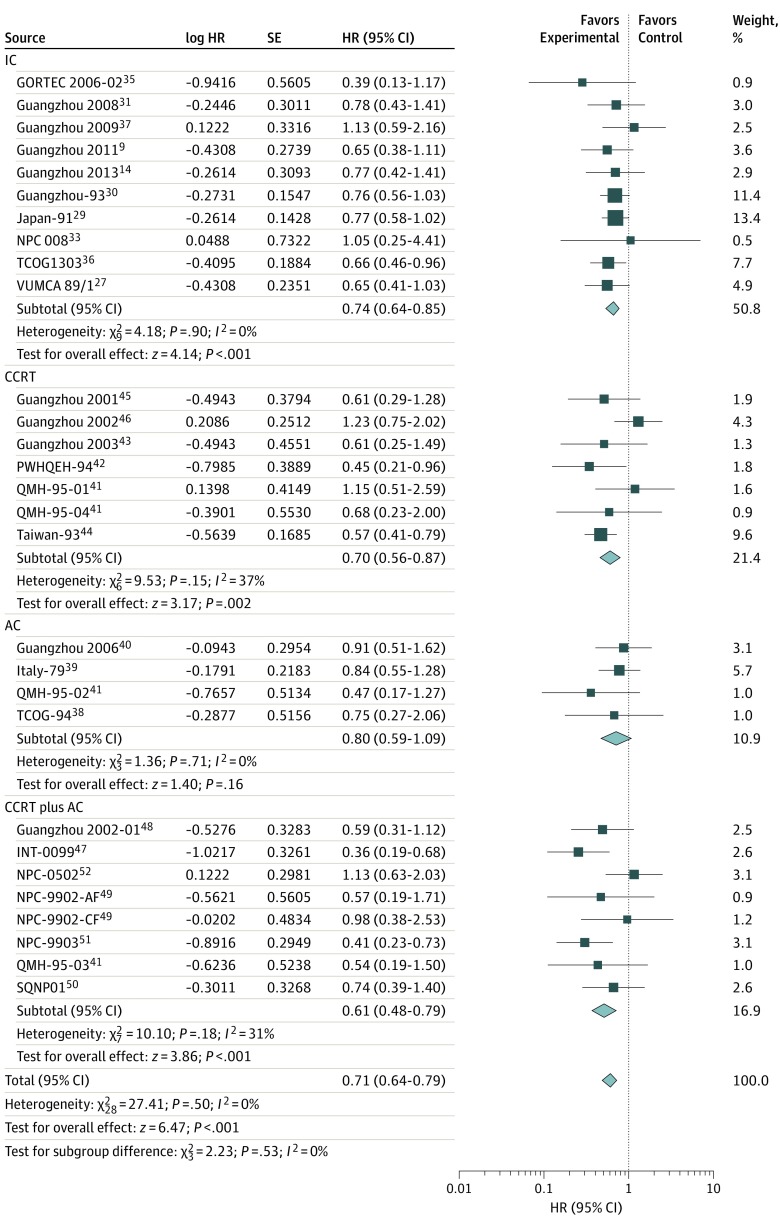
Locoregional Recurrence-Free Survival With Hazard Ratios (HRs) by Timing of Chemotherapy The center of each square is the HR for individual trial comparison, with the corresponding horizontal line showing the 95% CI. The size of the square is proportional to the number of locoregional recurrences from the trial. The center of the first 4 diamonds is the HR for different timings of chemotherapy, and the extremities are the 95% CIs. The center of the diamond at the bottom represents the overall pooled HR, with the extremities of the diamond showing the 95% CI. AC indicates adjuvant chemotherapy; AF, accelerated fractionation; CCRT, concomitant chemoradiotherapy; CF, conventional fractionation; GORTEC, Head and Neck Radiation Oncology Group; IC, induction chemotherapy; INT-0099, SWOG (Southwest Oncology Group)–coordinated Intergroup trial (also known as SWOG 8892); NPC, nasopharyngeal carcinoma; PWHQEH, Prince of Wales Hospital, Queen Elizabeth Hospital; QMH, Queen Mary Hospital; SQNP, Singapore Naso-Pharynx; TCOG, Taiwan Cooperative Oncology Group; and VUMCA, International Nasopharynx Cancer Study Group.

We collected data regarding the survival outcomes from 13 trials for IC, 6 trials for CCRT, 4 trials for AC, and 7 trials for CCRT plus AC. Of these, data regarding the LRFS for IC were unavailable from the Asian-Oceanian Clinical Oncology Association,^28^ Hellenic Cooperative Oncology Group,^32^ and Singapore 2004^34^ trials, and data regarding the PFS for CCRT were unavailable from the trial Guangzhou 2001.^45^ The results demonstrated that the addition of chemotherapy to radiotherapy was significantly associated with improved OS (HR, 0.76; 95% CI, 0.69-0.84; TSA-adjusted 95% CI, 0.69-0.84), PFS (HR, 0.72; 95% CI, 0.66-0.79; TSA-adjusted 95% CI, 0.66-0.79), DMFS (HR, 0.68; 95% CI, 0.62-0.75; TSA-adjusted 95% CI, 0.60-0.75), and LRFS (HR, 0.71; 95% CI, 0.64-0.79; TSA-adjusted 95% CI, 0.63-0.79) (Figure 1, Figure 2, Figure 3, and Figure 4). Low to moderate heterogeneity among trials was observed for OS (*I*^2^ = 34%, *P* = .03) and PFS (*I*^2^ = 36%, *P* = .02), whereas no significant heterogeneity was found among trials for LRFS (*I*^2^ = 21%, *P* = .14) and DMFS (*I*^2^ = 0%, *P* = .50).

Notably, the IC group was significantly associated with OS (HR, 0.84; 95% CI, 0.74-0.95; TSA-adjusted 95% CI, 0.74-0.95), PFS (HR, 0.73; 95% CI, 0.64-0.84; TSA-adjusted 95% CI, 0.64-0.84), DMFS (HR, 0.67; 95% CI, 0.59-0.78; TSA-adjusted 95% CI, 0.58-0.77), and LRFS (HR, 0.74; 95% CI, 0.64-0.85; TSA-adjusted 95% CI, 0.61-0.86) (Figure 1, Figure 2, Figure 3, and Figure 4). Furthermore, in the CCRT group, we observed significantly prolonged OS (HR, 0.66; 95% CI, 0.51-0.85; TSA-adjusted 95% CI, 0.51-0.85), PFS (HR, 0.73; 95% CI, 0.57-0.93; TSA-adjusted 95% CI, 0.57-0.93), DMFS (HR, 0.69; 95% CI, 0.56-0.85; TSA-adjusted 95% CI, 0.48-0.85), and LRFS (HR, 0.70; 95% CI, 0.56-0.87; TSA-adjusted 95% CI, 0.53-0.98) (Figure 1, Figure 2, Figure 3, and Figure 4). However, AC was not associated with additional survival benefit for OS (HR, 0.98; 95% CI, 0.78-1.23; TSA-adjusted 95% CI, 0.78-1.24), PFS (HR, 0.86; 95% CI, 0.70-1.07; TSA-adjusted 95% CI, 0.70-1.07), DMFS (HR, 0.84; 95% CI, 0.64-1.10; TSA-adjusted 95% CI, 0.58-1.22), or LRFS (HR, 0.80; 95% CI, 0.59-1.09; TSA-adjusted 95% CI, 0.59-1.09) (Figure 1, Figure 2, Figure 3, and Figure 4). The combined CCRT plus AC was associated with survival benefit compared with radiotherapy in terms of OS (HR, 0.63; 95% CI, 0.53-0.74; TSA-adjusted 95% CI, 0.54-0.74), PFS (HR, 0.64; 95% CI, 0.51-0.80; TSA-adjusted 95% CI, 0.51-0.80), DMFS (HR, 0.62; 95% CI, 0.51-0.76; TSA-adjusted 95% CI, 0.47-0.81), and LRFS (HR, 0.61; 95% CI, 0.48-0.79; TSA-adjusted 95% CI, 0.45-0.83) (Figure 1, Figure 2, Figure 3, and Figure 4).

For treatment outcomes of IC, CCRT, and CCRT plus AC, the cumulative *z* curve crossed the conventional boundaries (*z* = 1.96) and the monitoring boundaries for TSA, whereas for the outcomes of AC, the *z* curve did not cross the both boundaries. Hence, the TSA showed firm evidence on the treatment outcomes of IC, CCRT, and CCRT plus AC but absence of evidence on the treatment outcomes of AC. The required APIS of the TSA is shown in eFigures 5, 6, 7, and 8 in the Supplement.

### Subgroup Analyses

Subgroup analyses on the IC regimen found a significant association of IC plus radiotherapy with improvement in all end points compared with radiotherapy (OS: HR, 0.87; 95% CI, 0.74-1.03; PFS: HR, 0.73; 95% CI, 0.63-0.85; DMFS: HR, 0.71; 95% CI, 0.58-0.88; and LRFS: HR, 0.75; 95% CI, 0.62-0.90) and a significant association of IC plus CCRT in all end points compared with CCRT (OS: HR, 0.81; 95% CI, 0.68-0.96; PFS: HR, 0.73; 95% CI, 0.63-0.83; DMFS: HR, 0.64; 95% CI, 0.53-0.78; LRFS: HR, 0.73; 95% CI, 0.58-0.91) (eTable 4 in the Supplement). However, these associations were influenced by study center design, sample size, period of recruitment, tumor stage, and study bias. Single-center trials with sample sizes of 250 or less, period of recruitment longer than 5 years, tumor stage II, or high bias were less likely to find additional survival benefits. However, multicenter trials with sample sizes greater than 250, survival rate of 5 years or longer, median follow-up time longer than 60 months, and low bias have provided data suggestive of a benefit from adding IC to the treatment regimen for locoregionally advanced NPC. Subgroup analyses under various conditions demonstrated no additional survival benefit associated with AC in any end point. We observed persistent survival benefits associated with CCRT and CCRT plus AC vs radiotherapy for all end points analyzed. Low heterogeneity within subgroups was observed for any end point.

### Publication Bias

We found no evidence of publication bias based on visual inspection of funnel plots in terms of IC, CCRT, AC, and CCRT plus AC based on an analysis of pooled trials with sample sizes greater than 10 (eFigure 9 in the Supplement) or according to the Egger test or Begg test.

### Serious Adverse Events

eTable 5 in the Supplement lists the severe (grades 3-5) toxic effects of chemoradiotherapy. The severe toxic effects of the AC regimen could not be analyzed because of unavailable or inadequate data. Primary toxic effects included hematologic toxic effects and digestive system toxic effects. Both IC plus CCRT and CCRT plus AC were associated with the highest frequency of acute toxic effects. The late toxic effects were mainly associated with radiotherapy.

## Discussion

### Summary of Main Findings

This updated and comprehensive meta-analysis (comprising 28 RCTs with 8036 patients) of the role of chemotherapy regimens in NPC confirmed the benefits associated with the addition of chemotherapy to radiotherapy, including significant and clinically relevant improvements in all outcomes. The results of this study support the use of CCRT as the standard treatment for locoregionally advanced NPC, which was significantly associated with improvement in survival. The addition of IC but not AC to radiotherapy or CCRT could achieve prolonged OS, PFS, DMFS, and LRFS. The TSA provided firm evidence on the additional benefit associated with IC. However, the benefits associated with the addition of AC still lack evidence, which suggests more high-quality RCTs are needed.

### Comparison With Other Studies

Compared with the previous meta-analyses listed in eTable 3 in the Supplement, this study has analyzed more trials and patients and has included data on toxic effects in the analysis insofar as was possible. Although this meta-analysis is similar to the one by You et al,^53^ there are some differences. You et al^53^ found that CCRT plus AC was associated with a better survival benefit compared with CCRT and IC plus CCRT for LRFS, whereas our study found no additional benefit associated with AC plus CCRT for all end points. In addition, this updated meta-analysis answered a question about whether the current evidence is inconclusive and conducted subgroup analyses to identify sources of heterogeneity to interpret the inconsistent findings of previous trials.

Management of advanced NPC remains challenging for practitioners. Concurrent chemoradiotherapy has been adopted as the standard treatment for locoregionally advanced NPC, which is supported by previous meta-analyses (eTable 3 in the Supplement). Our study with TSA confirmed the association of CCRT with improvement in OS, PFS, DMFS, and LRFS compared with radiotherapy alone. Part of the current controversy regarding supportive evidence for combination treatment relates to the roles of IC and AC. Meta-analyses published in 2015 or earlier^8,54,55,56,57^ reported no significant differences between IC plus CCRT and CCRT with respect to OS and conflicting findings in PFS (eTable 3 in the Supplement). This finding might be because the meta-analyses included trials reported before 2013 but did not include trials using new IC regimens (eg, gemcitabine, cisplatin, and paclitaxel; cisplatin, fluorouracil, and docetaxel; and gemcitabine and cisplatin). Meta-analyses published after 2015^58,59,60,61^ indicated significant benefits associated with adding IC to CCRT in prolonging survival outcomes (eTable 3 in the Supplement). Therefore, optimizing the IC regimen may be another orientation currently and in the future. Subgroup analyses showed additional benefit associated with adding IC to CCRT in multicenter trials or trials with sample sizes greater than 250, survival rates of 5 years or longer, median follow-up longer than 5 years, or low risk of bias. In contrast, single-center trials or trials with small sample sizes, tumor stage II, or high risk of bias were not significant with respect to the additional benefits of IC. These key points may be why we found inconsistent findings in previous meta-analyses (listed in eTable 3 in the Supplement). The TSA provided sound evidence on the additional value of IC. However, patients with advanced NPC comprise many subgroups, and not all of them could benefit from additional IC. Epstein-Barr virus DNA and imaging biomarkers were incorporated as selection factors for clinical trials of IC to determine who could benefit from the treatment.^62^

In this meta-analysis, AC was not associated with any additional benefit in any of the end points, not only in the pooled analyses but also in the subgroup analyses. This finding was also supported by the preliminary results of meta-analyses (eTable 3 in the Supplement).^8,54,57,63,64^ Although some retrospective studies^65,66,67^ found an improvement in survival and fewer patients with distant metastases when 2 or more cycles of AC regimen were delivered, additional AC was poorly tolerated, with 55% to 75% adherence at best, and patients were at risk of more chemotherapy-related toxic effects.^15^ Although most trials used the cisplatin and fluorouracil AC regimen for advanced NPC, this combination perhaps benefitted only those with lower burden of distant tumor.^40^ Notably, only 4 trials^38,39,40,41^ investigated AC, of which 2 trials^38,39^ added AC to radiotherapy and 2 trials^40,41^ added AC to CCRT. The TSA produced an absence of evidence that AC alone could provide additional benefit. The required sample sizes ranged from 1698 to 2453 for the end points, which indicates that additional trials are needed.

### Strengths and Limitations

Our meta-analysis has several strengths. We performed a comprehensive search of several databases and sources to identify eligible trials. We adopted strict methods following the recommendations of the Cochrane Collaboration and PRISMA statement, including but not limited to a prepublished protocol, an up-to-date literature search and independent study selection, data extraction, and risk-of-bias assessment by at least 2 investigators. To our knowledge, this is the largest conventional meta-analysis. The large number of patients allowed for subgroup analyses to be performed with adequate power. We performed subgroup analyses in various aspects to find out the potential sources of heterogeneity and ensure the reliability and soundness of our findings. Moreover, we considered comprehensive time-to-event data of OS, PFS, DMFS, and LRFS to evaluate the benefits of IC, CCRT, AC, and CCRT plus AC regimens. When reporting an RCT with survival-type data, the appropriate summary statistics are the log HR and its variance. Hence, we used the outcome measure HR (calculated if unavailable) instead of the OR or relative risk to express the outcomes, which takes into account the number and timing of events and the time until last follow-up for each patient who has not experienced an event (ie, has been censored). Although our review uniquely aims to examine whether and when sufficient evidence of the additional survival benefit of IC and AC has been accrued, repeated meta-analyses with accumulating trial data could lead to random errors or false-positive results if multiple tests are not accounted for. We reduced the risk of random error in the updated meta-analysis by the TSA approach to increase the robustness of the analyses; to our knowledge, this method has not been used in existing meta-analyses on chemotherapy regimens for locoregionally advanced NPC.

Limitations of this study should be acknowledged. First, we could not evaluate the effects of various radiotherapy strategies, including 2-dimensional radiotherapy, 3-dimensional conformal radiotherapy, and intensity-modulated radiotherapy, on the heterogeneity among trials. Some previously published trials used outdated conventional or 2-dimensional radiotherapy. Second, unlike the individual patient data meta-analysis, we could not identify the interaction between treatment effect on survival end point and the timing of chemotherapy. Third, patients with stage II or World Health Organization type I cancer were included, but they represent few patients with NPC in both clinical practice and trials (Table).

## Conclusions

This updated meta-analysis with TSA confirmed the benefits associated with the addition of chemotherapy to radiotherapy for patients with locoregionally advanced NPC; the greatest benefit was found in those groups with concomitant administration, suggesting that CCRT should be the standard treatment. The addition of IC instead of AC to CCRT was associated with an additional survival benefit. However, the additional value of the AC regimen to CCRT needs further assessment.

## Notes

Supplement. **eTable 1.** Search Strategy **eTable 2.** Inclusion Criteria and Exclusion Criteria of Including Trials **eTable 3.** Comparisons Between the Meta-analyses **eFigure 1.** Process of Identification and Selection of Relevant Articles in This Meta-analysis **eFigure 2.** Country Distribution of the Included Trials **eFigure 3.** Risk of Bias Summary: Review Authors’ Judgements About Each Risk of Bias Item for Each Included Study **eFigure 4.** Risk of Bias Graph: Review Authors’ Judgements About Each Risk of Bias Item Presented as Percentages Across All Included Studies **eFigure 5.** Trial Sequential Analysis for Induction Chemotherapy Regimen **eFigure 6.** Trial Sequential Analysis for Concurrent Chemoradiotherapy Regimen **eFigure 7.** Trial Sequential Analysis for Adjuvant Chemotherapy Regimen **eFigure 8.** Trial Sequential Analysis for Concurrent Chemoradiotherapy Plus Adjuvant Chemotherapy Regimen **eTable 4.** Subgroup Analyses of IC, CCRT, AC, CCRT Plus AC on Various Conditions **eFigure 9.** Funnel Plots of Overall Survival (a), Progression-Free Survival (b), Distance Metastasis Free Survival (c), and Locoregional Recurrence Free Survival (d) **eTable 5.** Severe (Grades 3-5) Toxicities Related to Chemoradiotherapy Regimens
