# Error in Results

**DOI:** 10.1001/jamanetworkopen.2019.17197

**Published:** 2019-11-13

In the Original Investigation titled “Association of Chemoradiotherapy Regimens and Survival Among Patients With Nasopharyngeal Carcinoma: A Systematic Review and Meta-analysis,”^1^ published October 18, 2019, there was an error in which the data regarding distant metastasis–free survival (DMFS) and locoregional recurrence-free survival (LRFS) were switched in the last sentence of the first paragraph of the Primary Clinical End Points and Trial Sequence Analysis subsection of the Results. This sentence should have read, “Low to moderate heterogeneity among trials was observed for OS (*I*^2^ = 34%, *P* = .03) and PFS (*I*^2^ = 36%, *P* = .02), whereas no significant heterogeneity was found among trials for DMFS (*I*^2^ = 21%, *P* = .14) and LRFS (*I*^2^ = 0%, *P* = .50).” This article has been corrected.^1^

## References

[1] Zhang B, Li MM, Chen WH, Association of chemoradiotherapy regimens and survival among patients with nasopharyngeal carcinoma: a systematic review and meta-analysis*.* JAMA Netw Open. 2019;2(10):e1913619. doi:10.1001/jamanetworkopen.2019.13619 31626318 PMC6813597

