# Supplementary material for: Association of Chemoradiotherapy Regimens and Survival Among Patients With Nasopharyngeal Carcinoma: A Systematic Review and Meta-analysis
Source: JAMA Netw Open. 2019 Oct 18;2(10):e1913619. doi: 10.1001/jamanetworkopen.2019.13619 (PMC6813597; doi:10.1001/jamanetworkopen.2019.13619)
Supplement: Supplement. — eTable 1. Search Strategy eTable 2. Inclusion Criteria and Exclusion Criteria of Including Trials eTable 3. Comparisons Between the Meta-analyses eFigure 1. Process of Identification and Selection of Relevant Articles in This Meta-analysis eFigure 2. Country Distribution of the Included Trials eFigure 3. Risk of Bias Summary: Review Authors’ Judgements About Each Risk of Bias Item for Each Included Study eFigure 4. Risk of Bias Graph: Review Authors’ Judgements About Each Risk of Bias Item Presented as Percentages Across All Included Studies eFigure 5. Trial Sequential Analysis for Induction Chemotherapy Regimen eFigure 6. Trial Sequential Analysis for Concurrent Chemoradiotherapy Regimen eFigure 7. Trial Sequential Analysis for Adjuvant Chemotherapy Regimen eFigure 8. Trial Sequential Analysis for Concurrent Chemoradiotherapy Plus Adjuvant Chemotherapy Regimen eTable 4. Subgroup Analyses of IC, CCRT, AC, CCRT Plus AC on Various Conditions eFigure 9. Funnel Plots of Overall Survival (a), Progression-Free Survival (b), Distance Metastasis Free Survival (c), and Locoregional Recurrence Free Survival (d) eTable 5. Severe (Grades 3-5) Toxicities Related to Chemoradiotherapy Regimens [file jamanetwopen-2-e1913619-s001.pdf]

Supplementary Online Content

Zhang B, Li MM, Chen WH, et al. Association of chemoradiotherapy regimens and survival among patients with nasopharyngeal carcinoma: a systematic review and meta-analysis. *JAMA Netw Open*. 2019;2(10):e1913619. doi:10.1001/jamanetworkopen.2019.13619

eTable 1. Search Strategy

eTable 2. Inclusion Criteria and Exclusion Criteria of Including Trials

eTable 3. Comparisons Between the Meta-analyses

eFigure 1. Process of Identification and Selection of Relevant Articles in This Meta-analysis

eFigure 2. Country Distribution of the Included Trials

eFigure 3. Risk of Bias Summary: Review Authors' Judgements About Each Risk of Bias Item for Each Included Study

eFigure 4. Risk of Bias Graph: Review Authors' Judgements About Each Risk of Bias Item Presented as Percentages Across All Included Studies

eFigure 5. Trial Sequential Analysis for Induction Chemotherapy Regimen

eFigure 6. Trial Sequential Analysis for Concurrent Chemoradiotherapy Regimen

eFigure 7. Trial Sequential Analysis for Adjuvant Chemotherapy Regimen

eFigure 8. Trial Sequential Analysis for Concurrent Chemoradiotherapy Plus Adjuvant Chemotherapy Regimen

eTable 4. Subgroup Analyses of IC, CCRT, AC, CCRT Plus AC on Various Conditions

eFigure 9. Funnel Plots of Overall Survival (a), Progression-Free Survival (b), Distance Metastasis Free Survival (c), and Locoregional Recurrence Free Survival (d)

eTable 5. Severe (Grades 3-5) Toxicities Related to Chemoradiotherapy Regimens

This supplementary material has been provided by the authors to give readers additional information about their work.

© 2019 Zhang B et al. *JAMA Network Open*.

eTable 1: Search strategy

**PubMed**		
#1	Nasopharyngealneoplasms/drugtherapy [MAJR] OR nasopharyngealneoplasms/radiotherapy [MAJR]	2967
#2	random* OR ((Phase III) Fields: Title Word)	1276127
#3	clinical trial [Publication Type]	827493
#4	clinical trial, phase III [Publication Type] OR randomized controlled trial [Publication Type] OR meta-analysis [Publication Type]	586936
#5	#2 AND #3	502779
#6	#1 AND #5	143
#7	#1 AND #4	161
#8	#6 OR #7	175
**Web of Science Core Collection**		
#1	TS = (nasopharyn* OR cavum)	28412
#2	TS = (chemotherapy OR chemoradiation OR chemoradiotherapy OR radiochemotherapy OR radio-chemotherapy OR pharmacotherapy)	379962
#3	TS = (cancer*OR carcinoma* OR adenocarcinoma*OR malignan* OR tumor* OR tumour* OR neoplasm)	2851195
#4	TS = (random*)	1516158
#5	#1 AND #2 AND #3 AND #4	834
**EMBASE**		
#1	nasopharyn*: ab, ti OR cavum: ab, ti	45216
#2	chemotherapy: ab, ti OR chemoradiation: ab, ti OR chemoradiotherapy: ab, ti OR radiochemotherapy: ab, ti OR radio-chemotherapy: ab, ti OR pharmacotherapy: ab,ti	597193
#3	cancer*: ab, ti OR carcinoma*: ab, ti OR ‘adenocarcinoma*: ab, ti OR malignan*: ab, ti OR tumor*: ab, ti OR tumour*: ab, ti OR neoplasm: ab, ti	4082100
#4	random*: ab,ti	1406825
#5	#1 AND #2 AND #3 AND #4	563

© 2019 Zhang B et al. *JAMA Network Open*.

eTable 2: Inclusion criteria and exclusion criteria of including trials

Trial	Inclusion criteria	Exclusion criteria
Italy-79[39]<br>(Rossi, A. 1988)	Histopathologic diagnosis documented in the nasopharynx (at least with positive cytology in those patients where histology had been primarily applied to neck node metastases),undifferentiated or squamous-cell carcinoma, age <70 years, no evidence of distant metastatic disease, no previous therapy, no synchronous or previous neoplasm, performance status >70,absence of neuropsychiatric disorders, geographic accessibility,<br>adequate staging, expected cooperation for therapy and follow-up.	Not mentioned.
VUMCA 89/1[27]<br>(Cvitkovic 1996)	biopsy-proven UCNT (World Health Organization 2-3), age between 15-70, any T, N > 2, M0 (UICC 1987), performance status 0-2 (WHO). Prerandomization workup required CT scan of the nasopharynx, base of the skull, and cervical nodes, adequate (≥75 mL/min creatinin clearance) renal function, normal cardiac and hematological functions, and chest x-ray, bone scintigraphy, bone marrow biopsy, liver ultrasound and/or CT scan showing no evidence of distant metastases.	Not mentioned.
AOCOA[28]<br>(Chua D.T. 1998)	Ho's Stage III/IV disease, or any stage with a neck lymph Node ≥3 cm in greatest dimension. Only patients with histologically proven undifferentiated or poorly differentiated carcinoma were eligible. Patients should not have had previous treatment for their disease and were required to have a pretreatment Eastern Cooperative Oncology Group performance status of ≤2.Adequate bone marrow reserve was required, with a leukocyte count of at least 4000/uL and a platelet count of at least 100,000/uL. Serum bilirubin less than 1.5 mg/dL, serum creatinine less than 1.5 mg/dL, and creatinine clearance greater than 60 mL/min were also required. Patients also had to have a normal electrocardiograph.	Patients with a history of cardiac or renal disease.
INT-0099[47]<br>(Al-Sarraf M1998)	biopsy-proven stage III and IV cancers of the nasopharynx. without evidence of systemic metastasis (MO). There could be no history of previous radiotherapy or chemotherapy, and no history of previous cancer except for carcinoma-in-situ of the cervix or basal cell or squamous carcinoma of the skin. There must be no plan for resection following radiotherapy except for neck dissection for persistent neck nodes after completion of radiotherapy. Patients were required to have the following laboratory values: WBC count ≥4,000/uL, platelet count≥100,000/uL, creatinine concentration≤1.6mg/dL, and/or creatinine clearance ≥60 mL/min. Patients were also required to have a Southwest Oncology Group (SWOG) performance score of 0 to 2.	Not mentioned.
Guangzhou-93[30]<br>(Ma, J. 2001)	histologically proven NPC; stage III or IV disease; no evidence of systemic metastasis (M0); and World Health Organization (WHO) performance status of 0 to 2. There could be no history of radiotherapy or chemotherapy, and no history of prior malignancy except for nonmelanoma cancers of the skin or in situ cervical carcinoma. Patients were required to have adequate hematologic (leukocyte count ≥4,000/mm^3, platelet count ≥100,000/mm^3), hepatic (serum bilirubin level <1.5mg/dL), and renal (serum creatinine level, 1.5 mg/dL and/or 24-hour urinary creatinine clearance ≥60 mL/min) functions. A normal ECG was also required.	Patients with a history of cardiac or renal disease.
TCOG-94[38]<br>(Chi KH. 2002)	biopsy-proven carcinoma of nasopharynx, Stage IV (UICC/AJCC 1992) disease (including T4N0–1M0 and TN2–3M0), and performance status 0–2 (WHO). Patients were required to have adequate bone marrow function (WBC [white blood cells] ≥4,000/L, platelet count ≥100,000/L, hemoglobin ≥10.5 mg%), renal function (serum creatinine ≤1.5 mg/dL), and liver function (total bilirubin ≤2.0 mg/dL). Patients were allowed no previous history of chemotherapy or radiotherapy.	Patients with serology evidence of hepatitis B surface antigen.
Japan-91[29]<br>(Hareyama,M2002)	age 10–70 years, no detectable distant metastases (M0), a Karnofsky index >50, and a creatinine clearance >30.Pretreatment studies included an endoscopic examination of the nasopharynx; a biopsy of the tumor; and computed tomography scans of the nasopharynx, base of the skull, and cervical lymph nodes. Patients also were required to have adequate renal function (>30 mL per minute creatinine clearance); normal cardiac and hematologic functions; and a chest radiograph, bone scintigraphy, and liver computed tomography scan showing no evidence of distant metastases. Magnetic resonance imaging of the nasopharynx also was performed in most patients.	Not mentioned.
Taiwan-93[44]<br>(Lin, J. C.2003)	Patients with biopsy-proven NPC and stage III to IV (M0) disease according to the 1992 American Joint Committee on Cancer staging system. Patients could have no history of previous RT or chemotherapy, and no history of previous cancer except for carcinoma-insitu of the cervix or nonmelanoma cancers of the skin. Karnofsky performance status≥60%; WBC count greater than 4,000/L and	Not mentioned.

© 2019 Zhang B et al. *JAMA Network Open*.

	platelet count greater than 100,000/L; serum creatinine level less than 1.6 mg/dL; normal liver function with total bilirubin less than 2.5 mg/dL; and no detectable distant metastasis.	
QMH-95[41] (Kwong, D. 2004)	Patients with histologically proven, previously untreated NPC with Ho's stage T3 or N2/N3 disease or with any lymph nodes ≥4 cm and no distant metastases at diagnosis. WBC counts of at least 4,000/L, platelet counts of at least 100,000/L, creatinine clearance of at least 60 mL/min, normal liver function, and chest x-ray.	Not mentioned.
PWHQEH-94[42] (Chan, A. T.2005)	Patients with biopsy-proven, previously untreated NPC with Ho's N2 or N3 stage or N1 stage with nodal size ≤4 cm， Patients were required to have no prior history of cancer except for basal cell or squamous cell carcinoma of the skin. WBC count ≥4,000/L, platelet count ≥100,000/L, creatinine clearance ≥50 mL/min, and no evidence of systemic metastases.	Not mentioned.
SQNP01[50] (Wee, J. 2005)	All patients who had American Joint Committee on Cancer / International Union Against Cancer (1997) T3-4NxM0 or TxN2-3M0 previously untreated NPC with WHO type II or III histology; an Eastern Cooperative Oncology Group performance status of 0 or 1; and adequate bone marrow, renal, and hepatic functions.	previous treatment for NPC, presence of distant metastasis, and other concomitant malignant disease.
NPC 008[33] (Hui, E. P.2009)	Patients were eligible if they had biopsy-proven, previously untreated, locoregionally advanced NPC of International Union Against Cancer 1997stages III to IVB. Other eligibility criteria included assessable disease, Eastern Cooperative Oncology Group performance status grade 0 or 1, age of at least 18 years, adequate bone marrow reserve (WBC count and platelet count of at least the lower limit of normal) and renal function (serum creatinine <1.5 the upper limit of normal or creatinine clearance ≥50 mL/min), and absence of hypercalcemia or second malignancy.	Not mentioned.
Guangzhou2003[43] (Chen QY 2011)	biopsy-proven World Health Organization (WHO) types II–III NPC , Stage II disease (T1-2N1M0 or T2N0M0 with parapharyngeal space involvement), between ages 18 and 70 years, adequate hematologic function (white blood cell counts ≥ 4000/μL and platelet counts ≥100000/μL), adequate renal function (creatinine clearance ≥50 mL/min), adequate hepatic function (serum bilirubin level <1.5 mg/dL), and satisfactory performance status (a score of 0 or 1 using the Eastern Cooperative Oncology Group System).	previous treatment of NPC, the presence of a distant metastasis, or prior malignancy (except carcinoma in situ of the cervix or basal/squamous cell carcinoma of the skin).
NPC-9902[49] (Lee, A. W. 2011)	Biopsy-proven nonkeratinizing (type 2.1) or undifferentiated (type 2.2) carcinoma of the nasopharynx by the World Health Organization classification. Stage T3-4N0-1M0 by the staging system of American Joint Committee on Cancer Staging System and the International Union Against Cancer , fifth edition (AJCC/UICC-5); Adequate hematologic function: total leukocyte count (white blood cell count≥4,000/L and platelet ≥100,000/L; Adequate renal function: creatinine clearance >60 mL/min; and Satisfactory performance status: <2 by the Eastern Cooperative Oncology Group System.	keratinizing squamous cell carcinoma or adenocarcinoma, age ≥70 years, pregnancy or lactation, history of previous treatment or prior malignancy (except adequately treated carcinoma in situ of the cervix, or basal/squamous cell carcinoma of the skin).
HeCOG[32] (Fountzilas, G2012)	biopsy-proven, previously untreated WHO type I, II or III NPC; Age ≥15 years; stage IIB–IVB according to the American Joint Committee on Staging of Cancer classification (AJCC 2002); Measurable or evaluable disease; no other primary tumors; performance status(PS) of 0-2 according to the Eastern Cooperative Oncology Group scale(ECOG); adequate bone marrow, hepatic, renal and pulmonary function; adequate nutritional status to tolerate protocol treatment and adequate mental status to follow instructions, keep appointments and provide written informed consent.	Not mentioned.
Guangzhou2002-01[48] (Chen Y 2013)	Patients between ages 16 and 70 years with stage III to IVB (according to the fifth edition of the American Joint Commission on Cancer [AJCC] cancer staging manual), histologically proven, nonkeratinizing NPC. an Eastern Cooperative Oncology Group system (ECOG) performance status between 0 and 2 and adequate bone marrow, liver, and renal function.	Pregnancy or lactation, a history of renal disease, unstable cardiac disease requiring treatment, any previous chemotherapy or radiotherapy, and prior malignancy.
Guangzhou 2001[45] (Wu, X. 2013)	Patients were evaluated using the 1997 American Joint Committee on Cancer (AJCC)/International Union Against Cancer staging system. Patients were required to have had histologic proof of WHO subtype I, II, or III NPC. Disease stage had to be N2/N3 and any T or T3/T4 and any N without evidence of distant metastases (M0). Patients were at least 18 years of age, had an Eastern Cooperative Oncology Group performance status of2,and provided written informed consent. Adequate renal, hepatic ,and bone marrow function (WBC count ≥4,000/L and platelet count ≥100,000/L) and normal ECG were also required.	Not mentioned.
Singapore 2004[34] (Tan, T.2015)	All patients newly diagnosed with World Health Organization type 2 or 3 NPC, Union for International Cancer Control (1997) stage T3-4NxM0 or TxN2-3M0, at least 18 years of age, and having an Eastern Cooperative Oncology Group	Patients who had uncontrolled hypercalcemia, a second malignancy, serious active infections, other serious concomitant systemic disorders incompatible

© 2019 Zhang B et al. *JAMA Network Open*.

	performance status of 0 or 1 and adequate bone marrow, renal, and hepatic function.	with the study, previous treatments including chemotherapy or RT, or were pregnant, lactating, reproductive females not implementing adequate contraceptive measures, or hepatitis B carriers.
Guangzhou 2002[46] (Huang, P. Y.2015)	pathologically diagnosed nonkeratinizing or undifferentiated carcinoma of the nasopharynx (World Health Organization [WHO] types II or III), age of 18–65 years, a performance status score according to the Eastern Cooperative Oncology Group System of 0–2 points, Chinese 1992 staging system stage III or IV disease, and adequate hematologic (white blood cell [WBC] count ≥4.0 × 10^9/L, platelet[PLT] ≥100×10^9/L), hepatic (total bilirubin[TBIL], alanine aminotransferase [ALT] <2 times the normal value), and renal function (creatinine [Cr] <1.5 times the normal value).	pregnancy or lactation, prior malignancy, history of previous anticancer therapy, uncontrolled infection, and unsuitability for chemotherapy due to impaired kidney, liver, lung, or heart function.
Guangzhou2008[31] (Cao SM 2017)	Previously untreated, biopsy-proven WHO types II-IIINPC; Stage III-IVB disease, excluding T3N0-1 (UICC/AJCC 6th edition);Between 18 and 60 years old; Adequate bone marrow, liver and renal function; Satisfactory performance status: a score of 0 or 1 using the Eastern Cooperative Oncology Group System; Patients provided signed informed consent.	Evidence of relapse or distant metastasis; History of prior malignancy or previous treatment for NPC; The presence of uncontrolled life-threatening diseases; The investigator considered the patient unable to complete the study.
Guangzhou2006[40] (Chen, L. 2017)	Eligible patients were aged 18-70 years with non-metastatic, histologically proven non-keratinising stage III or IV NPC, except T3-4N0 (6th American Joint Commission on Cancer staging system).	Not mentioned.
Guangzhou 2009[37] (Jin, Y. N. 2017)	presence of histologically confirmed NPC; no evidence of distant metastases; receiving intensity-modulated radiotherapy (IMRT); treated with concurrent or/and neoadjuvant chemotherapy; presence of undetectable(0 copy/ml) pEBV DNA; And absence of secondary malignancy, pregnancy, or lactation.	Not mentioned.
NPC-9903[51] (Lee, A. W. 2017)	Eligible patients were those with histologically confirmed nonkeratinizing (differentiated or undifferentiated) carcinoma of the nasopharynx classified by the World Health Organization system and T1-4N2-3M0 disease (T = NPC tumor stage; N = nodal stage; M = evidence of distant metastases) classified by the staging criteria of the 5th edition of the American Joint Committee on Cancer Staging System and the International Union Against Cancer. performance status of 2 or lower by the Eastern Cooperative Oncology Group System and adequate hematologic (total leukocyte count≥4000/μL; platelet count≥100000/μL) and renal function (creatinine clearance≥60 mL/min).	age of 70 years or older, keratinizing squamous cell carcinoma or adenocarcinoma, pregnancy or lactation, history of previous treatment, or prior malignancy (except for adequately treated carcinoma in situ of the cervix, or basal or squamous cell carcinoma of the skin).
NPC-0502[52] (Chan, A. T 2018)	Eligible patients with histologically confirmed NPC of Union for International Cancer Control stage IIB to IVB, adequate organ function, and no locoregional disease or distant metastasis.	Not mentioned.
TCOG1303[36] (Hong, R. 2018)	Patients with histologically proved stage IVA or IVB NPC (fifth edition of the AJCC/UICC staging system, 1997), age <70 years, an Eastern Cooperative Oncology Group performance status≤1, normal hemogram, renal, and liver function.	Not mentioned.
GORTEC 2006-02[35] (Frikha, M.2018)	histological WHO type 2 or 3, stage T2b, T3,T4 and/or N1-N3, M0, PS 0–1 along with haematological, renal, cardiac and liver functions.	Not mentioned.
Guangzhou 2013[14] (Zhang Y2019)	an age between 18 and 64 years; histologic confirmation of nonkeratinizing nasopharyngeal carcinoma; no previous treatment for cancer; nondistant metastatic, newly diagnosed stage III to IVB disease (excluding subgroups of patients with low risk of metastasis; i.e., those with bulky primary tumor with no nodal involvement) that was staged according to the American Joint Committee on Cancer– Union for International Cancer Control 7th edition stage-classification system; a Karnofsky performance-status score of at least 70 (on a scale from 0 to 100, with lower scores indicating greater disability); and adequate hematologic, renal, and hepatic function.	receipt of treatment with palliative intent; a history of cancer; receipt of previous treatment (radiotherapy, chemotherapy, or surgery [except diagnostic procedures]) to the nasopharynx or neck; lactation or pregnancy; or severe coexisting illness.
Guangzhou2011[9] (Li WF2019)	patients were those with previously untreated, histologically confirmed, non-keratinizing, locoregionally advanced NPC (stage III-IVB except T3-4NO; 7th American Joint Commission on Cancer staging system). Patients had to be 18-59 years old with Karnofsky scores of at least 70, and adequate bone marrow, liver, and renal function.	Treatment with palliative intent, a history of previous radiotherapy, chemotherapy, or surgery (except diagnostic) to the primary tumor or nodes, previous malignancy, pregnancy or lactation, or any severe coexisting disease.

© 2019 Zhang B et al. *JAMA Network Open*.

eTable 3: Comparisons between the meta-analyses

Study	Classification	Inclusion period	No. of patients	Stage	WHO type	Group	OS	PFS	DMFS	RFS	Conclusions
Huncharek, M. 2002	conventional	1966-2001	1582	III, IV	I, II, III	CCRT group vs RT	OR 0.80 [0.63, 1.02]	OR 0.60 [0.49,0.73]	NA	NA	The addition of chemotherapy to standard radical radiation therapy for locoregionally advanced nasopharyngeal cancer increases both disease-free/progression-free and overall survival by 19 to 40% at 2 to 4 years after treatment, depending on the endpoint of interest.
Langendijk, J.A. 2004	conventional	1975-2000	2450	III, IV	I, II, III	IC group vs RT<br>CCRT group vs RT<br>AC group vs RT	HR 0.87 [0.72, 1.04]<br>HR 0.48 [0.32, 0.72]<br>HR 0.99 [0.71, 1.36]	NA	RR 0.67 [0.53, 0.83]<br>RR 0.70 [0.54, 0.92]<br>RR 0.89 [0.64, 1.26]	RR 0.74 [0.60, 0.91]<br>RR 0.47 [0.33, 0.67]<br>RR 0.79 [0.55, 1.14]	The results of this study indicate that concomitant chemotherapy in addition to radiation is probably the most effective way to improve OS in NPC.
Chua, D.T. 2005	conventional	1989-1994	784	III, IV	I, II, III	IC+RT vs RT	HR 0.83 [0.66, 1.03]	NA	NA	HR 0.79 [0.65, 0.96]	The addition of cisplatin-based induction chemotherapy to RT was associated with a modest but significant decrease in relapse and improvement in disease-specific survival in advanced stage NPC. However, there was no improvement in overall survival.
Baujat, B. 2006	conventional	~2003	1753	I, II, III, IV	I, II, III	IC+RT+/-AC vs RT<br>CCRT+/-AC vs RT<br>AC group vs RT	HR 0.99 [0.80, 1.21]<br>HR 0.60 [0.48, 0.76]<br>HR 0.97 [0.68, 1.38]	HR 0.82 [0.68, 0.97]<br>HR 0.63 [0.51, 0.78]<br>HR 0.90 [0.67, 1.20]	NA	NA	Chemotherapy led to a small but significant benefit for overall survival and event-free survival. This benefit was essentially observed when chemotherapy was administered concomitantly with radiotherapy.
Zhang, L. 2010	prospective	1994-2005	1608	II, III, IV	I, II, III	CCRT vs RT<br>CCRT+AC vs RT	RR 0.66 [0.48, 0.92]<br>RR 0.83 [0.63, 1.09]	NA	RR 0.71 [0.51, 0.99]<br>RR 0.71 [0.54, 0.92]	NA	This is the first meta-analysis of CCRT vs. RT alone in NPC treatment which included studies only done in endemic area. The results confirmed that CCRT was more beneficial compared with RT alone. However, the relative benefit of CCRT in endemic population might be less than that from previous meta-analyses.
Liang, Z.G. 2012	conventional	~2012	793	II, III, IV	I, II, III	CCRT+AC vs CCRT	RR 1.02 [0.89, 1.15]	NA	RR 0.95 [0.80, 1.13]	NA	Compared with concurrent chemoradiotherapy alone, concurrent chemotherapy followed by adjuvant chemotherapy did not improve prognosis. More toxicity was found during adjuvant chemotherapy.
Liang, Z.G. 2013	conventional	~2012	1096	II, III, IV	I, II, III	IC+CCRT vs CCRT+/-AC	RR 0.99 [0.72, 1.36]	RR 0.37 [0.20, 0.69]	RR 0.98 [0.75, 1.27]	NA	Compared with the control group, induction chemotherapy followed by concurrent chemoradiotherapy was well tolerated but could not significantly improve prognosis in terms of overall survival, loco-regional failure-free survival or distant metastasis failure-free survival.
OuYang, P.Y. 2013	conventional	NA	2605	II, III, IV	NA	NACT vs no NACT<br>AC vs no AC	HR 0.82 [0.69, 0.98]<br>HR 1.04 [0.79, 1.37]	NA	RR 0.69 [0.56, 0.84]<br>RR 0.93 [0.65, 1.33]	RR 0.90 [0.66, 1.22]<br>RR 0.71 [0.53, 0.96]	NACT can effectively enhance OS and reduce DMR, not LRR in NPC. And AC only helps to better control locoregional recurrence of NPC.

© 2019 Zhang B et al. *JAMA Network Open*.

Blanchard, P. 2015	IPD	~2010	4806	II, III, IV	I, II, III	IC vs control<br>AC vs control<br>CCRT vs control<br>CCRT+AC vs control	HR 0.96 [0.80, 1.16]<br>HR 0.87 [0.68, 1.12]<br>HR 0.80 [0.70, 0.93]<br>HR 0.65 [0.56, 0.76]	HR 0.81 [0.69, 0.95]<br>HR 0.80 [0.64, 1.00]<br>HR 0.81 [0.71, 0.92]<br>HR 0.62 [0.53, 0.72]	NA	NA	Our results confirm that the addition of concomitant chemotherapy to radiotherapy significantly improves survival in patients with locoregionally advanced nasopharyngeal carcinoma. To our knowledge, this is the first analysis that examines the effect of concomitant chemotherapy with and without adjuvant chemotherapy as distinct groups. Further studies on the specific benefits of adjuvant chemotherapy after concomitant chemoradiotherapy are needed.
Chen,Y.P.2015	network	~2014	1988	II, III, IV	II, III, IV	NACT+RT vs RT<br>CCRT vs RT<br>NACT+CCRT vs CCRT	HR 0.85 [0.72, 1.04]<br>HR 0.69 [0.51, 0.91]<br>HR 0.73 [0.40, 1.23]	NA	RR 0.72 [0.58, 0.88]<br>RR 0.75 [0.55, 0.97]<br>RR 0.54 [0.27, 0.94]	RR 0.75 [0.61, 0.93]<br>RR 0.96 [0.60, 1.42]<br>RR 1.71 [0.94, 2.84]	NACT+CCRT is associated with reduced distant failure as compared with CCRT alone, and whether the additional NACT can improve survival for locoregionally advanced NPC should be further explored. Optimizing regimens and identifying patients at high risk of metastasis may enhance the efficacy of NACT+CCRT.
Chen,Y.P.2014	network	NA	2144	II, III, IV	NA	CCRT+AC vs CCRT<br>CCRT+AC vs RT<br>CCRT vs RT	HR 0.86 [0.60, 1.16]<br>HR 0.59 [0.48, 0.71]<br>HR 0.69 [0.50, 0.92]	NA	HR 0.86 [0.62, 1.16]<br>HR 0.64 [0.50, 0.81]<br>HR 0.76 [0.56, 0.97]	HR 0.72 [0.43, 1.15]<br>HR 0.56 [0.36, 0.81]<br>HR 0.80 [0.51, 1.12]	No significant improvement was found following CCRT+AC compared with CCRT alone. Whether the omission of additional AC can reduce toxic effects without adversely affecting survival in patients with locoregionally advanced NPC should be further explored, in addition to the precise patient status that would benefit from AC following CCRT.
Song,Y.2015	conventional	~2015	798	III, IV	NA	IC+CCRT vs CCRT	HR 0.52 [0.21, 1.29]	HR 0.66 [0.49, 0.90]	HR 0.60 [0.39, 0.98]	NA	Induction chemotherapy could significantly reduce the hazard of progression and distant metastasis in LANPC on the basis of concurrent chemoradiotherapy, but do less with the hazard of overall death and loco-regional failure.
He, X.2015	conventional	NA	1277	NA	NA	IC+RT vs RT	OR 0.84 [0.64, 1.05]	NA	OR 0.61 [0.51, 0.73]	OR 0.65 [0.50, 0.84]	Neoadjuvant chemotherapy followed by radiation can decrease the risk of recurrence and metastasis but not improve the 5 years overall survival and 5 years disease free survival compared to radiotherapy alone in the patients with locally advanced nasopharyngeal carcinoma.
Wang, Y.S.2015	conventional	NA	2576	III, IV	NA	CCRT vs RT	OR 0.53 [0.44, 0.64]	NA	OR 0.55 [0.43, 0.71]	NA	The results suggested that CCRT was more beneficial when compared with RT alone in locoregionally advanced NPC patients. Further study is needed to perform to confirm this effect.
Yan, M.2015	network	~2014	5576	II, III, IV	I, II, III	CRT-A vs CRT<br>N-CRT vs CRT<br>CRT-A vs N-CRT	HR 0.98 [0.71, 1.34]<br>HR 1.03 [0.69, 1.47]<br>HR 0.96 [0.64, 1.48]	NA	NA	NA	Adjuvant chemotherapy does not appear to improve survival following CRT. The efficacies of CRT, CRT-A and N-CRT all appeared to be similar. Further studies are warranted to determine the value of additional chemotherapy phases in specific patient subgroups.
Wang, M.2016	conventional	~2015	2215	III, IV	II, III	NACT+CCRT vs CCRT+/-AC	HR 0.64 [0.49, 0.84]	HR 0.68 [0.56, 0.81]	NA	NA	Our meta-analysis confirmed that the addition of NACT to CCRT significantly improved PFS and OS versus CCRT with or without AC for locoregionally advanced nasopharyngeal

© 2019 Zhang B et al. *JAMA Network Open*.

											carcinoma. These results may alter the standard of care - CCRT with or without AC, for locoregionally advanced nasopharyngeal carcinoma.
Yu, H.L.2016	network	~2015	2626	II, III, IV	NA	IC+CCRT vs RT<br>CCRT+AC vs RT<br>CCRT vs RT	HR 0.43 [0.18, 0.81]<br>HR 0.63 [0.45, 0.83]<br>HR 0.65 [0.45, 0.91]	NA	RR 0.42 [0.18, 0.82]<br>RR 0.64 [0.48, 0.83]<br>RR 0.77 [0.55, 1.05]	RR 1.31 [0.51, 2.89]<br>RR 0.55 [0.36, 0.82]<br>RR 0.73 [0.45, 1.15]	For locally advanced nasopharyngeal cancer, no significant differences in the treatment efficacies of CCRT, IC + CCRT, and CCRT + AC were found, with the exception of a marginally significant improvement in distant control observed following IC + CCRT compared with CCRT alone.
He, J.2017	network	NA	10081	II, III, IV	I, II, III, IV	CCRT vs control<br>CCRT+AC vs control<br>IC+CCRT vs control<br>IC vs control<br>IC+AC vs control	HR 0.70 [0.59, 0.85]<br>HR 0.64 [0.52, 0.79]<br>HR 0.74 [0.57, 0.96]<br>HR 0.80 [0.65, 0.98]<br>HR 0.54 [0.31, 0.93]	NA	NA	NA	In view of survival rate and complete response, the NMA results revealed that C, C + A and C + N showed excellent efficacy. As a result, these 3 therapies were supposed to be considered as the first-line treatment according to this NMA.
Ribassin-Majed, L 2017	network	~2010	5144	III, IV	II, III, IV	IC+RT vs RT<br>IC+CRT vs RT<br>CRT vs RT<br>CRT+AC vs RT<br>RT+AC vs RT<br>IC+RT+AC vs RT	HR 0.92 [0.75, 1.12]<br>HR 0.81 [0.63, 1.04]<br>HR 0.77 [0.64, 0.92]<br>HR 0.65 [0.56, 0.75]<br>HR 0.96 [0.71, 1.29]<br>HR 0.87 [0.58, 1.30]	HR 0.78 [0.66, 0.93]<br>HR 0.68 [0.54, 0.85]<br>HR 0.77 [0.65, 0.91]<br>HR 0.62 [0.54, 0.71]<br>HR 0.84 [0.63, 1.11]<br>HR 0.83 [0.59, 1.17]	NA	NA	The addition of AC to CRT achieved the highest survival benefit and consistent improvement for all end points. The addition of IC to CRT achieved the highest effect on distant control.
Xu, C.2017	prospective	~2016	2138	II	I, II, III	CRT vs RT	HR 0.67 [0.45, 0.98]	NA	HR 0.83 [0.52, 1.31]	HR 0.61 [0.46, 0.80]	In the treatment of patients with stage II NPC, CRT was better than 2D-RT alone with significant benefit in LRRFS. IMRT alone was superior to CRT with equivalent survival outcomes and fewer grade 3-4 acute toxicities.
You, R.2017	network	~2017	7094	II, III, IV	I, II, III	IC+RT vs RT<br>RT+AC vs RT<br>IC+RT+ AC vs RT<br>CCRT vs RT<br>CCRT+AC vs RT<br>IC+CCRT vs RT	HR 0.78 [0.64, 0.96]<br>HR 1.07 [0.79, 1.44]<br>HR 0.95 [0.60, 1.52]<br>HR 0.71 [0.58, 0.86]<br>HR 0.69 [0.58, 0.82]<br>HR 0.58 [0.46, 0.73]	HR 0.72 [0.63, 0.83]<br>HR 0.84 [0.63, 1.12]<br>HR 0.84 [0.59, 1.20]<br>HR 0.73 [0.64, 0.84]<br>HR 0.66 [0.58, 0.75]<br>HR 0.55 [0.47, 0.64]	HR 0.61 [0.49, 0.75]<br>HR 0.88 [0.58, 1.35]<br>HR 1.06 [0.66, 1.70]<br>HR 0.70 [0.57, 0.85]<br>HR 0.58 [0.48, 0.70]<br>HR 0.48 [0.37, 0.61]	HR 0.78 [0.62, 0.99]<br>HR 0.64 [0.38, 1.08]<br>HR 0.53 [0.31, 0.89]<br>HR 0.77 [0.59, 1.00]<br>HR 0.56 [0.44, 0.72]<br>HR 0.64 [0.48, 0.86]	IC-CRT should be the most suitable regimen for loco-regionally advanced NPC in the IMRT era.
Chen, Y.P.2018	IPD	~2017	1193	III, IV	II, III	IC+CCRT vs CCRT	HR 0.75 [0.57, 0.99]	HR 0.70 [0.56, 0.86]	NA	NA	This IPD pooled analysis demonstrates the superiority of additional IC over CCRT alone in locoregionally advanced NPC, with the survival benefit mainly associated with improved distant control.
He, Y.2018	conventional	~2017	1142	III, IV	NA	CCRT vs RT	RR 0.44 [0.26, 0.77]	NA	NA	NA	Compared with IMRT alone, CCRT provided survival benefit with acceptable toxicity in patients with LANPC. However, we need multicenter randomized controlled trials and long-term follow-up to evaluate the eventual efficacy and toxicity of concurrent chemotherapy plus IMRT.
Liu, M.2018	network	~2018	3248	III, IV	NA	CCRT vs RT<br>CCRT+AC vs RT<br>IC+CCRT vs RT	HR 0.76 [0.63, 0.93]<br>HR 0.66 [0.57, 0.77]<br>HR 0.52 [0.37, 0.74]	NA	HR 0.68 [0.53, 0.87]<br>HR 0.58 [0.47, 0.71]<br>HR 0.40 [0.27, 0.58]	HR 0.80 [0.57, 1.12]<br>HR 0.59 [0.45, 0.78]<br>HR 0.54 [0.33, 0.89]	IC+CCRT may be a better and more promising treatment strategy for advanced NPC; however, head-to-head

© 2019 Zhang B et al. *JAMA Network Open*.

											randomized trials comparing IC-CCRT with CCRT-AC are warranted.
Tan, T.H.2018	conventional	1996-2017	2802	II, III, IV	NA	IC+CCRT vs CCRT	HR 0.77 [0.60, 0.98]	HR 0.69 [0.57, 0.84]	HR 0.63 [0.47, 0.83]	NA	IC delays disease progression and improves survival significantly for LA-NPC treated with CCRT, and was associated with more toxicity. There were no divergent results between RCTs and OBS. IC followed by CCRT can be considered one of the standard treatment options for LA-NPC.
Wang, S.F.2018	conventional	~2018	3038	II	I, II, III	CRT vs RT	RR 1.04 [1.01, 1.06]	RR 1.05 [1.00, 1.10]	RR 1.00 [0.97, 1.03]	RR 1.05 [1.02, 1.07]	Compared with conventional RT alone, CRT could significantly improve patients' prognoses in terms of OS, PFS, and LRFS for stage-II NPC, but not DMFS, and CRT can provide greater benefits from concurrent chemotherapy than neoadjuvant chemotherapy. With IMRT, the stage-II NPC patients did not benefit from the addition of chemotherapy.

Abbreviations: WHO, World Health Organization; OS, overall survival; PFS, progression-free survival; DMFS, distance metastasis free survival; RFS, recurrence free survival; IC, induction chemotherapy; CCRT, concurrent chemoradiotherapy; AC, adjuvant chemotherapy; NACT, neoadjuvant chemotherapy; N-CRT, neoadjuvant chemotherapy-concurrent chemoradiotherapy; CRT-A, concurrent chemoradiotherapy-adjuvant chemotherapy RT, radiotherapy; HR, hazard ratio RR, risk ratio OR, odds ratio; NA, not available.

© 2019 Zhang B et al. *JAMA Network Open*.

eFigure 1. Process of identification and selection of relevant articles in this meta-analysis.

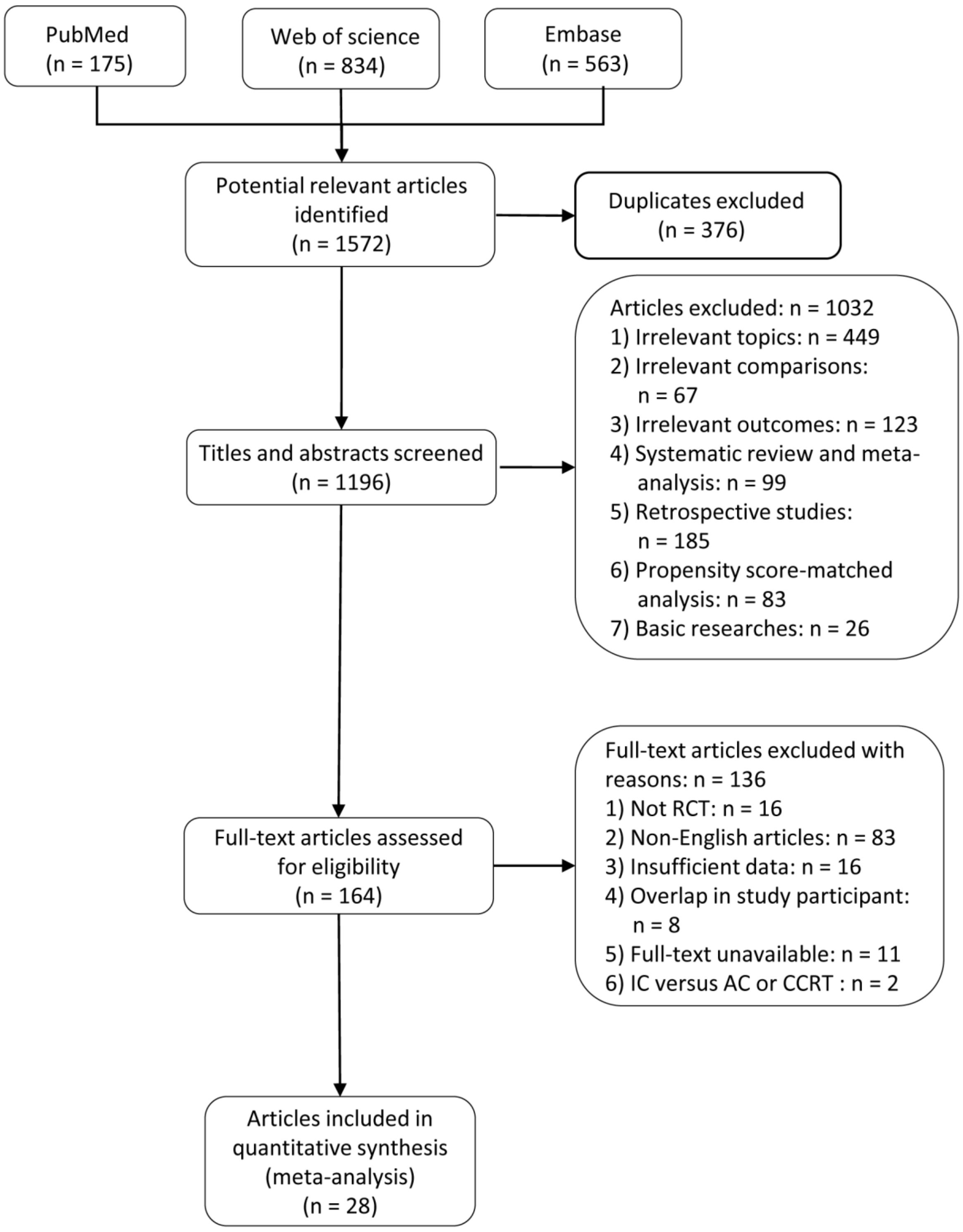

© 2019 Zhang B et al. *JAMA Network Open*.

eFigure 2. Country distribution of the inclued trials. The word map shows that most of trials were performed in China (column), and some trials were international (purple arc curve).

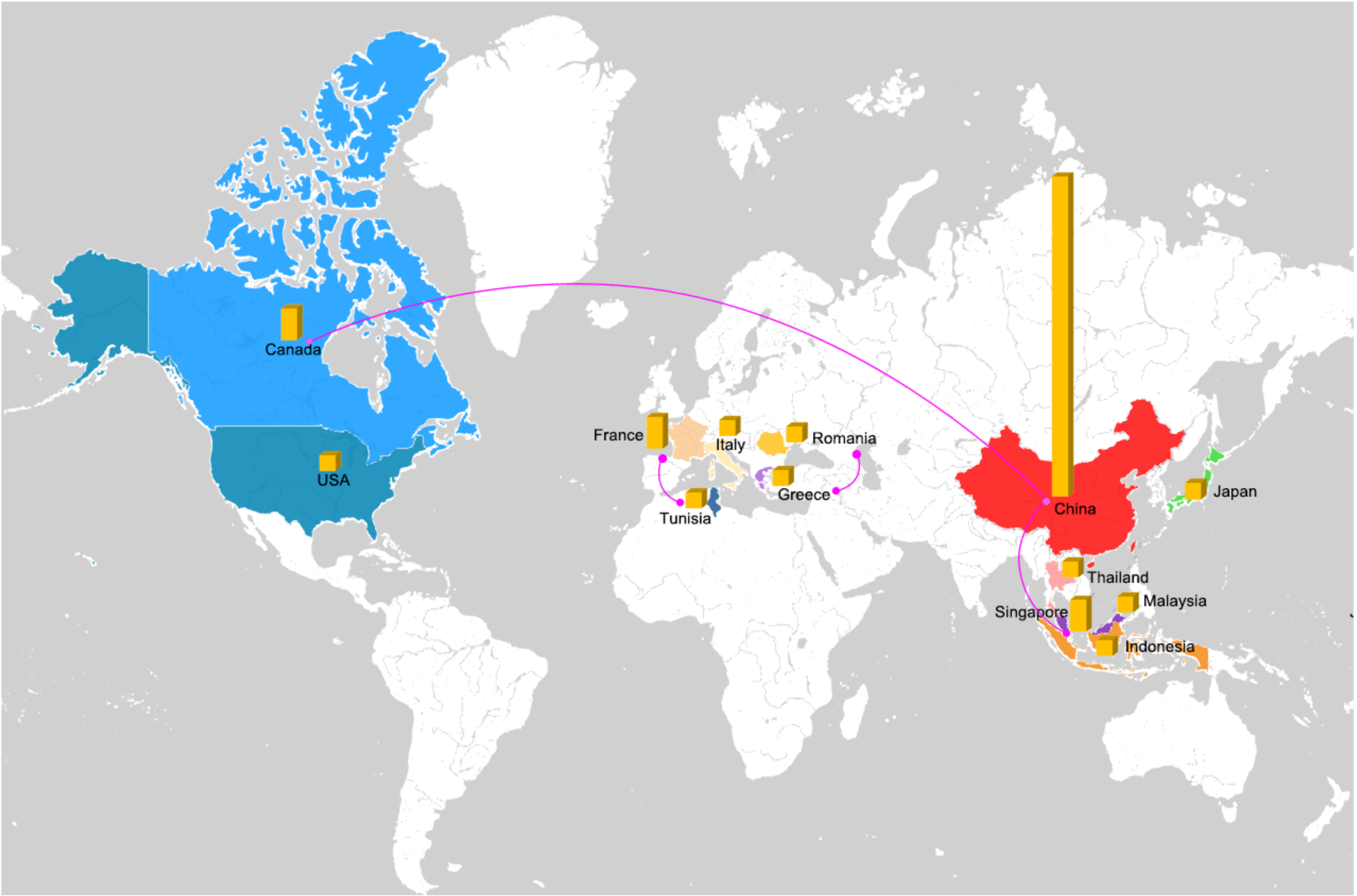

© 2019 Zhang B et al. *JAMA Network Open*.

eFigure 3. Risk of bias summary: review authors' judgements about each risk of bias item for each included study.

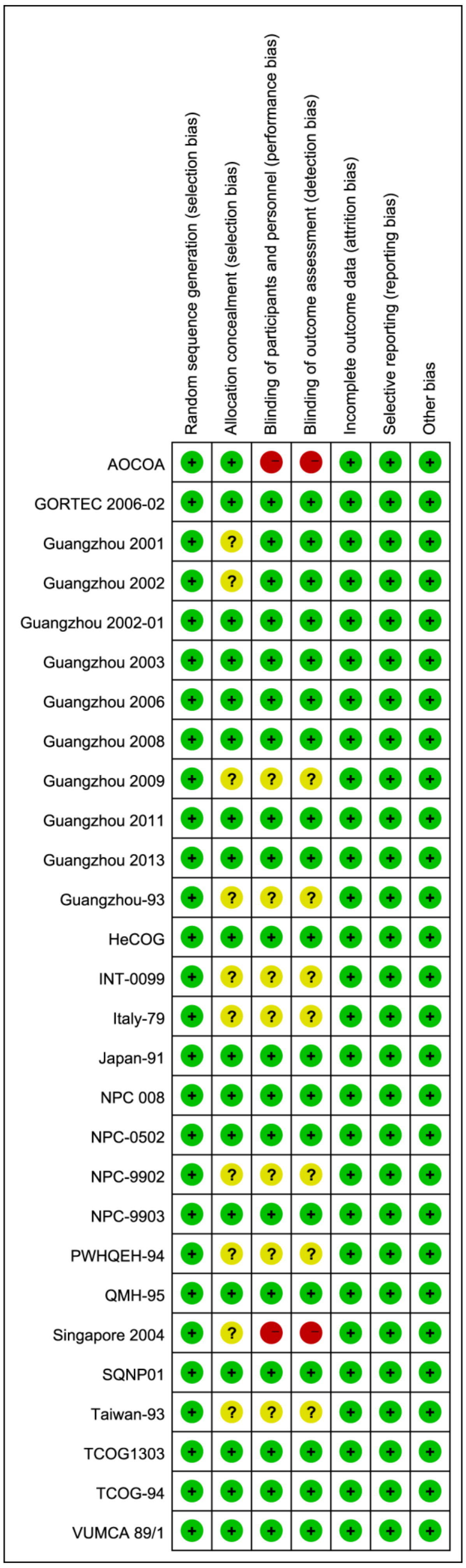

© 2019 Zhang B et al. *JAMA Network Open*.

eFigure 4. Risk of bias graph: review authors' judgements about each risk of bias item presented as percentages across all included studies.

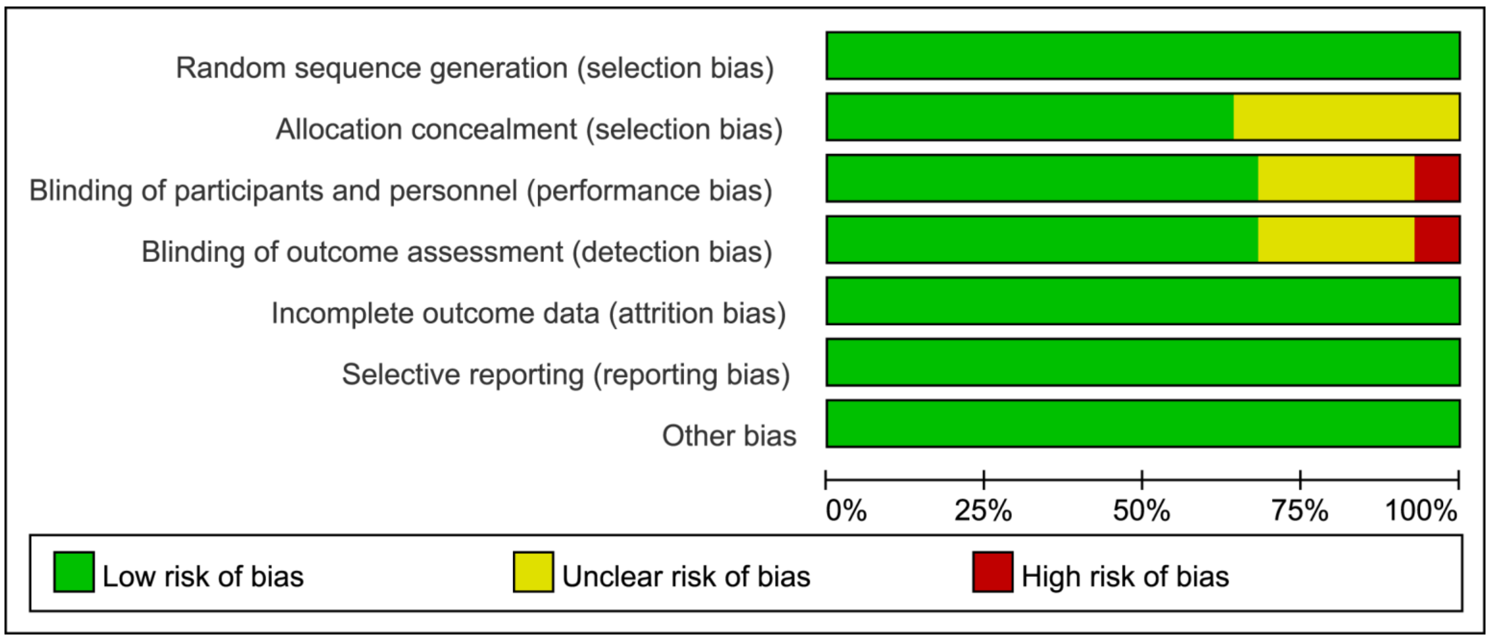

© 2019 Zhang B et al. *JAMA Network Open*.

eFigure 5. Trial sequential analysis for induction chemotherapy regimen.

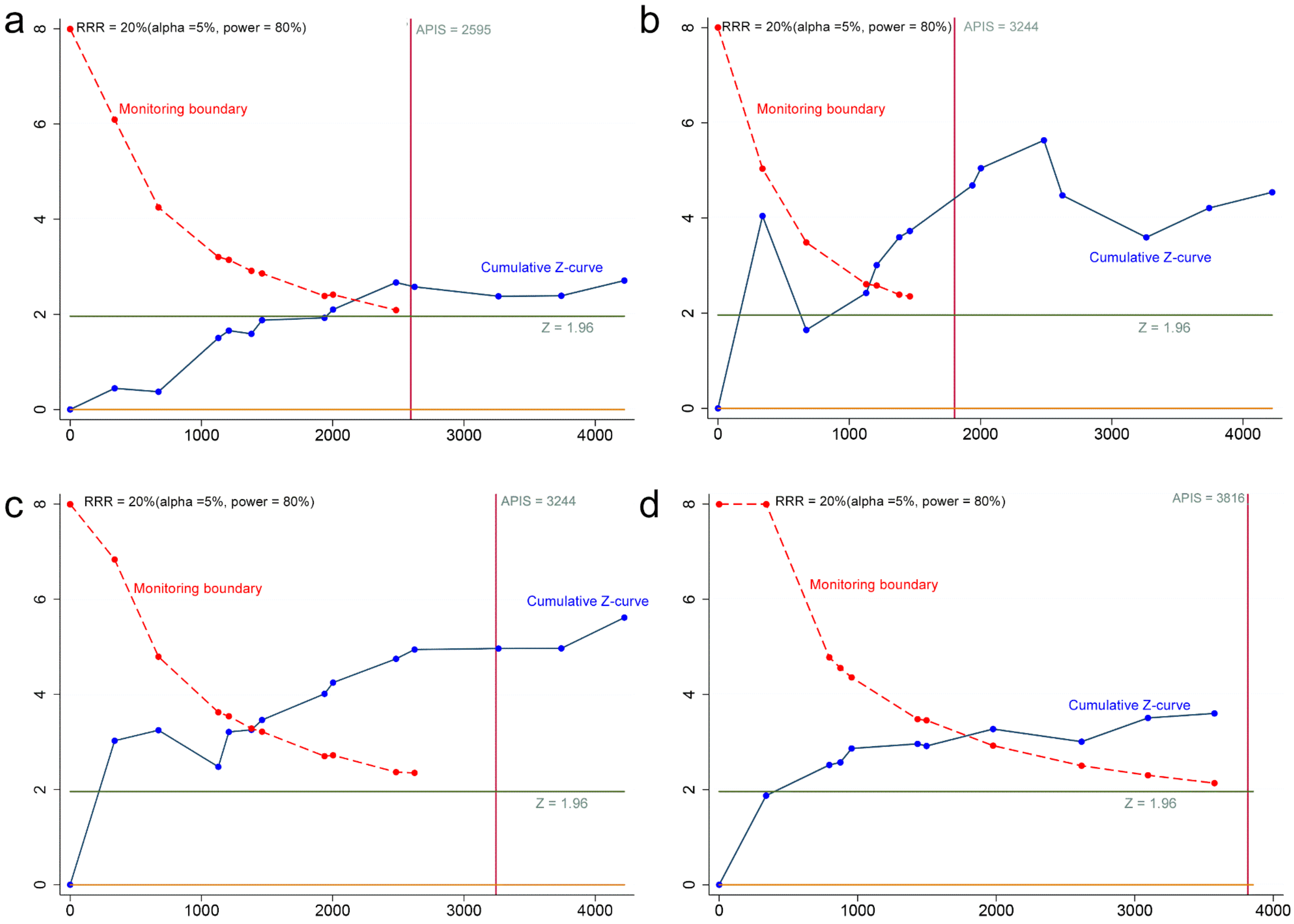

(a) For OS, a APIS of 2595 patients was calculated based on an anticipated RRR of 20% (average survival rate = 75%, loss follow-up = 2.0%, α=0.05 (two-sided), β=0.20 (power 80%)). The blue cumulative z-curve was constructed using a random-effects model and crossed the conventional boundary (Z=1.96, green line) and monitoring boundary (red dotted line) for futility.(b) For PFS, a APIS of 3244 patients was calculated based on an anticipated RRR of 20% (average survival rate = 64%, loss follow-up = 2.0%, α=0.05 (two-sided), β=0.20 (power 80%)). The blue cumulative z-curve was constructed using a fixed-effects model and crossed the conventional boundary(Z=1.96, green line) and monitoring boundary (red dotted line) for futility. (c) For DMFS, a APIS of 3244 patients was calculated based on an anticipated RRR of 20% (average survival rate = 80%, loss follow-up = 2.0%, α=0.05 (two-sided), β=0.20 (power 80%)). The blue cumulative z-curve was constructed using a fixed-effects model and crossed the conventional boundary(Z=1.96, green line) and monitoring boundary (red dotted line) for futility. (d) For LRFS, a APIS of 3816 patients was calculated based on an anticipated RRR of 20% (average survival rate = 83%, loss follow-up = 2.0%, α=0.05 (two-sided), β=0.20 (power 80%)). The blue cumulative z-curve was constructed using a fixed-effects model and crossed the conventional boundary (Z=1.96, green line) and monitoring boundary (red dotted line) for futility. Abbreviations: OS = overall survival; APIS = a priori information size; RRR = relative risk reduction; PFS = progression-free survival; DMFS = distance metastasis free survival; LRFS = locoregional recurrence free survival.

© 2019 Zhang B et al. *JAMA Network Open*.

eFigure 6. Trial sequential analysis for concurrent chemoradiotherapy regimen.

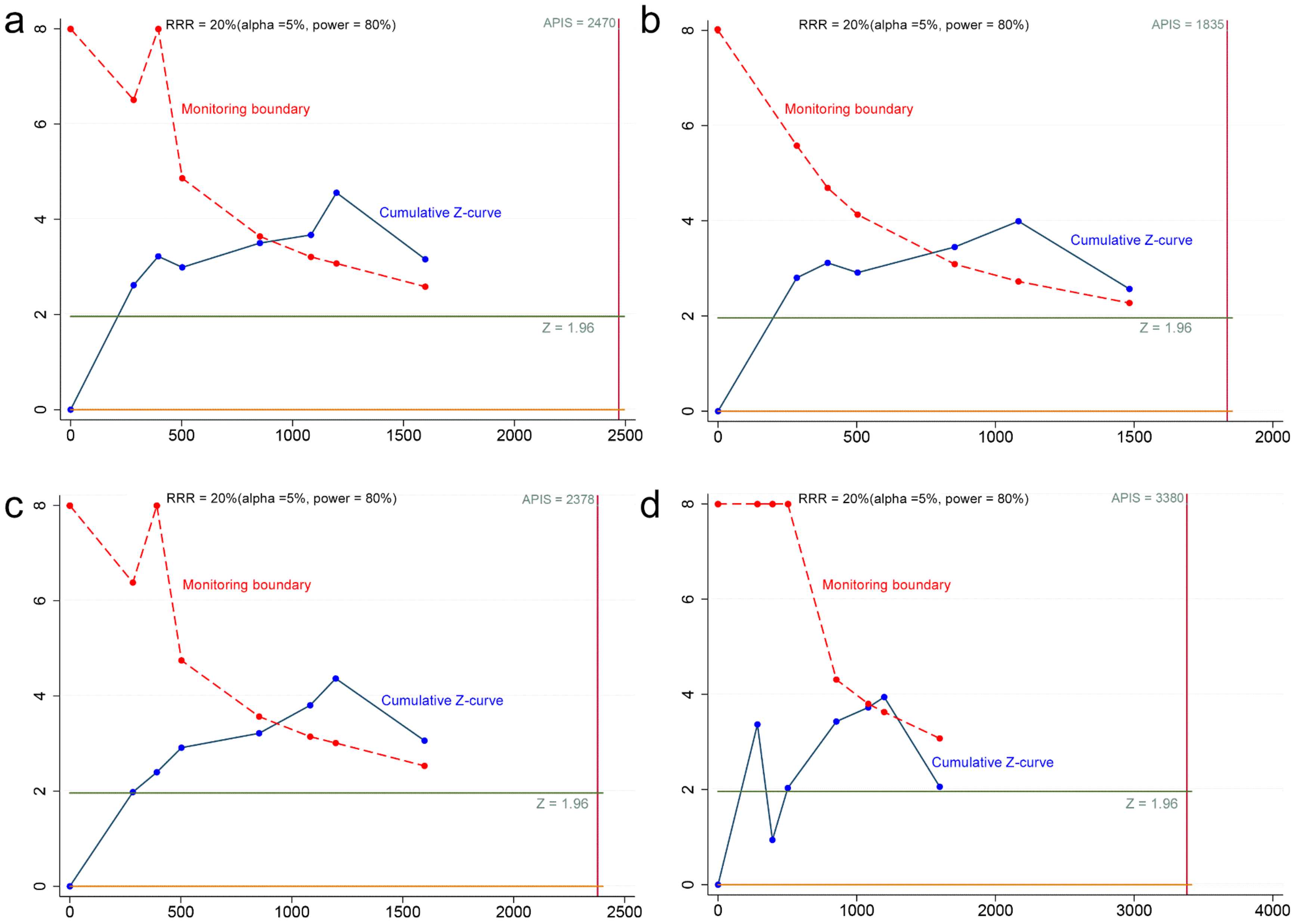

(a) For OS, a APIS of 2470 patients was calculated based on an anticipated RRR of 20% (average survival rate = 74%, loss follow-up = 0.7%, α=0.05 (two-sided), β=0.20 (power 80%)). The blue cumulative z-curve was constructed using a random-effects model and crossed the conventional boundary(Z=1.96, green line) and monitoring boundary (red dotted line) for futility. (b) For PFS, a APIS of 1835 patients was calculated based on an anticipated RRR of 20% (average survival rate = 65%, loss follow-up = 0.7%, α=0.05 (two-sided), β=0.20 (power 80%)). The blue cumulative z-curve was constructed using a fixed-effects model and crossed the conventional boundary(Z=1.96, green line) and monitoring boundary (red dotted line) for futility. (c) For DMFS, a APIS of 2378 patients was calculated based on an anticipated RRR of 20% (average survival rate = 73%, loss follow-up = 0.7%, α=0.05 (two-sided), β=0.20 (power 80%)). The blue cumulative z-curve was constructed using a fixed-effects model and crossed the conventional boundary(Z=1.96, green line) and monitoring boundary (red dotted line) for futility. (d) For LRFS, a APIS of 3380 patients was calculated based on an anticipated RRR of 20% (average survival rate = 81%, loss follow-up = 0.7%, α=0.05 (two-sided), β=0.20 (power 80%)). The blue cumulative z-curve was constructed using a fixed-effects model and crossed the both conventional boundary(Z=1.96, green line) and monitoring boundary (red dotted line) for futility. Abbreviations: OS = overall survival; APIS = a priori information size; RRR = relative risk reduction; PFS = progression-free survival; DMFS = distance metastasis free survival; LRFS = locoregional recurrence free survival.

© 2019 Zhang B et al. *JAMA Network Open*.

eFigure 7. Trial sequential analysis for adjuvant chemotherapy regimen.

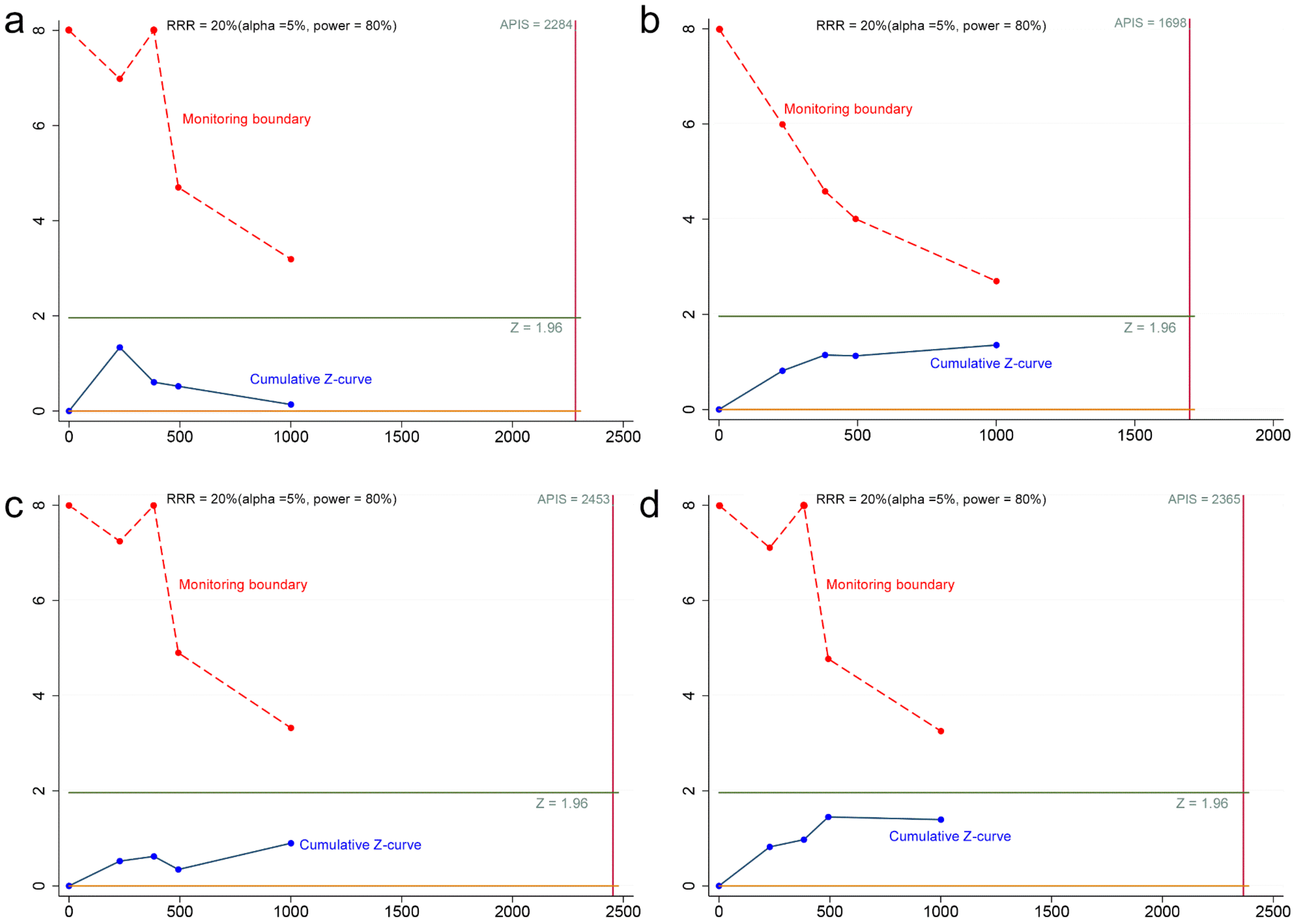

(a) For OS, a APIS of 2284 patients was calculated based on an anticipated RRR of 20% (average survival rate = 71%, loss follow-up = 3.5%, α=0.05 (two-sided), β=0.20 (power 80%)). The blue cumulative z-curve was constructed using a random-effects model and did not crossed the conventional boundary(Z=1.96, green line) and monitoring boundary (red dotted line) for futility. (b) For PFS, a APIS of 1698 patients was calculated based on an anticipated RRR of 20% (average survival rate = 61%, loss follow-up = 3.5%, α=0.05 (two-sided), β=0.20 (power 80%)). The blue cumulative z-curve was constructed using a fixed-effects model and did not crossed the conventional boundary(Z=1.96, green line) and monitoring boundary (red dotted line) for futility. (c) For DMFS, a APIS of 2453 patients was calculated based on an anticipated RRR of 20% (average survival rate = 73%, loss follow-up = 3.5%, α=0.05 (two-sided), β=0.20 (power 80%)). The blue cumulative z-curve was constructed using a fixed-effects model and did not crossed the conventional boundary(Z=1.96, green line) and monitoring boundary (red dotted line) for futility. (d) For LRFS, a APIS of 2365 patients was calculated based on an anticipated RRR of 20% (average survival rate = 72%, loss follow-up = 3.5%, α=0.05 (two-sided), β=0.20 (power 80%)). The blue cumulative z-curve was constructed using a fixed-effects model and did not crossed the conventional boundary(Z=1.96, green line) and monitoring boundary (red dotted line) for futility. Abbreviations: OS = overall survival; APIS = a priori information size; RRR = relative risk reduction; PFS = progression-free survival; DMFS = distance metastasis free survival; LRFS = locoregional recurrence free survival.

© 2019 Zhang B et al. *JAMA Network Open*.

eFigure 8. Trial sequential analysis for concurrent chemoradiotherapy plus adjuvant chemotherapy regimen.

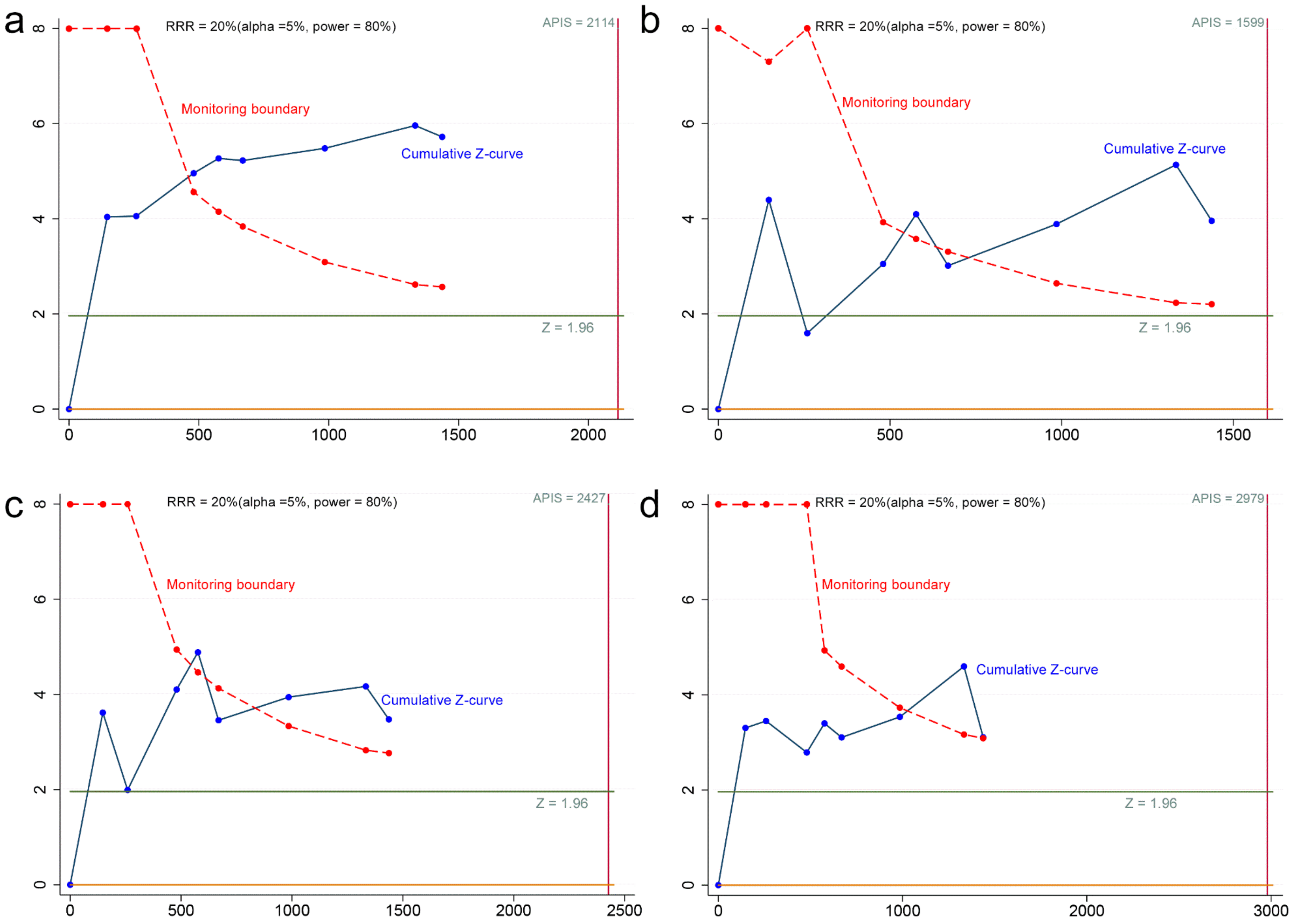

(a) For OS, a APIS of 2114 patients was calculated based on an anticipated RRR of 20% (average survival rate = 69%, loss follow-up = 3.0%, α=0.05 (two-sided), β=0.20 (power 80%)). The blue cumulative z-curve was constructed using a random-effects model and crossed the conventional boundary(Z=1.96, green line) and monitoring boundary (red dotted line) for futility. (b) For PFS, a APIS of 1599 patients was calculated based on an anticipated RRR of 20% (average survival rate = 59%, loss follow-up = 3.0%, α=0.05 (two-sided), β=0.20 (power 80%)). The blue cumulative z-curve was constructed using a fixed-effects model and crossed the conventional boundary(Z=1.96, green line) and monitoring boundary (red dotted line) for futility. (c) For DMFS, a APIS of 2427 patients was calculated based on an anticipated RRR of 20% (average survival rate = 73%, loss follow-up = 3.0%, α=0.05 (two-sided), β=0.20 (power 80%)). The blue cumulative z-curve was constructed using a fixed-effects model and crossed the conventional boundary(Z=1.96, green line) and monitoring boundary (red dotted line) for futility. (d) For LRFS, a APIS of 2979 patients was calculated based on an anticipated RRR of 20% (average survival rate = 78%, loss follow-up = 3.0%, α=0.05 (two-sided), β=0.20 (power 80%)). The blue cumulative z-curve was constructed using a fixed-effects model and crossed the conventional boundary(Z=1.96, green line) and monitoring boundary (red dotted line) for futility. Abbreviations: OS = overall survival; APIS = a priori information size; RRR = relative risk reduction; PFS = progression-free survival; DMFS = distance metastasis free survival; LRFS = locoregional recurrence free survival.

eTable 4. Subgroup analyses of IC, CCRT, AC, CCRT plus AC on various conditions

Subgroup	trials	OS	PFS	DMFS	LRFS
**Chemotherapy regimens**					
IC					
IC plus RT vs RT	4	0.87 (0.74-1.03)	0.73 (0.63-0.85)	0.71 (0.58-0.88)	0.75 (0.62-0.90)
IC plus CCRT vs CCRT	9	0.81 (0.68-0.96)	0.73 (0.63-0.83)	0.64 (0.53-0.78)	0.73 (0.58-0.91)
CCRT					
CCRT vs RT	5	0.62 (0.49-0.77)	0.66 (0.53-0.83)	0.57 (0.44-0.75)	0.60 (0.47-0.78)
IC plus CCRT vs IC plus RT	1	0.95 (0.72-1.26)	1.01 (0.77-1.34)	1.02 (0.72-1.45)	1.23 (0.75-2.02)
CCRT plus AC vs RT plus AC	1	0.47 (0.22-1.02)	0.65 (0.36-1.18)	0.65 (0.37-1.14)	0.68 (0.23-2.00)
AC					
RT plus AC vs RT	2	1.11 (0.82-1.50)	0.83 (0.61-1.14)	0.80 (0.54-1.17)	0.82 (0.55-1.22)
CCRT plus AC vs CCRT	2	0.83 (0.59-1.18)	0.89 (0.67-1.19)	0.88 (0.60-1.30)	0.77 (0.47-1.27)
CCRT + AC					
CCRT + AC vs RT	7a	0.63 (0.53-0.74)	0.62 (0.53-0.73)	0.62 (0.51-0.76)	0.61 (0.48-0.79)
**Study centre design**					
Single centre					
IC	5/3*	0.87 (0.72-1.05)	0.89 (0.73-1.09)	0.77 (0.59-1.01)	0.82 (0.63-1.08)
CCRT	4/3¶	0.75 (0.61-0.91)	0.82 (0.67-1.01)	0.73 (0.59-0.92)	0.74 (0.58-0.94)
AC	1	0.86 (0.36-2.06)	0.93 (0.47-1.82)	1.40 (0.53-3.66)	0.47 (0.17-1.27)
CCRT plus AC	3	0.63 (0.48-0.83)	0.65 (0.52-0.82)	0.58 (0.43-0.79)	0.64 (0.42-0.97)
Multicentre					
IC	8/7*	0.82 (0.70-0.95)	0.68 (0.60-0.76)	0.64 (0.54-0.75)	0.71 (0.60-0.84)
CCRT	2	0.64 (0.46-0.88)	0.68 (0.51-0.90)	0.52 (0.32-0.86)	0.51 (0.29-0.91)
AC	3	0.99 (0.78-1.25)	0.86 (0.68-1.07)	0.80 (0.60-1.07)	0.85 (0.61-1.18)
CCRT + AC	4	0.63 (0.51-0.76)	0.60 (0.48-0.74)	0.65 (0.50-0.85)	0.60 (0.44-0.82)
**Publish year**					
Before 2015 (including 2015)					
IC	7	0.88 (0.76-1.02)	0.76 (0.66-0.87)	0.70 (0.58-0.84)	0.75 (0.62-0.90)
CCRT	6	0.71 (0.60-0.85)	0.77 (0.65-0.91)	0.69 (0.56-0.85)	0.70 (0.56-0.87)
AC	3	1.08 (0.81-1.43)	0.85 (0.64-1.13)	0.86 (0.60-1.23)	0.76 (0.53-1.10)
CCRT plus AC	5	0.58 (0.48-0.70)	0.60 (0.49-0.72)	0.54 (0.42-0.69)	0.58 (0.42-0.79)
After 2015					
IC	6	0.78 (0.64-0.94)	0.70 (0.60-0.81)	0.65 (0.53-0.80)	0.72 (0.58-0.90)
CCRT	0	none	none	none	none
AC	1	0.83 (0.57-1.21)	0.88 (0.64-1.21)	0.81 (0.53-1.24)	0.91 (0.51-1.62)

© 2019 Zhang B et al. *JAMA Network Open*.

CCRT plus AC	2	0.74 (0.56-0.98)	0.68 (0.52-0.90)	0.80 (0.58-1.11)	0.68 (0.45-1.02)
**Sample size**					
> 250					
IC	8/7❖	0.84 (0.74-0.96)	0.72 (0.64-0.80)	0.68 (0.59-0.80)	0.73 (0.62-0.87)
CCRT	3	0.78 (0.64-0.95)	0.81 (0.67-0.97)	0.81 (0.64-1.03)	0.68 (0.53-0.89)
AC	1	0.83 (0.57-1.21)	0.88 (0.64-1.21)	0.81 (0.53-1.24)	0.91 (0.51-1.62)
CCRT plus AC	2	0.68 (0.54-0.87)	0.61 (0.48-0.77)	0.68 (0.51-0.91)	0.48 (0.31-0.74)
≤250					
IC	5/3❖	0.81 (0.60-1.10)	0.78 (0.59-1.02)	0.62 (0.44-0.88)	0.75 (0.57-0.98)
CCRT	3/2❁	0.53 (0.37-0.76)	0.65 (0.45-0.93)	0.48 (0.33-0.69)	0.74 (0.48-1.14)
AC	3	1.08 (0.81-1.43)	0.85 (0.64-1.13)	0.86 (0.60-1.23)	0.76 (0.53-1.10)
CCRT plus AC	5a	0.58 (0.47-0.72)	0.63 (0.51-0.78)	0.58 (0.44-0.76)	0.69 (0.51-0.94)
**Period of recruitment**					
>5					
IC	4/3✧	0.91 (0.72-1.14)	0.72 (0.59-0.87)	0.74 (0.58-0.94)	0.74 (0.60-0.91)
CCRT	3	0.59 (0.42-0.83)	0.65 (0.48-0.87)	0.60 (0.43-0.85)	0.63 (0.47-0.85)
AC	1	0.86 (0.36-2.06)	0.93 (0.47-1.82)	1.40 (0.53-3.66)	0.47 (0.17-1.27)
CCRT plus AC	4	0.57 (0.45-0.73)	0.60 (0.47-0.76)	0.56 (0.41-0.75)	0.67 (0.48-0.94)
≤5					
IC	9/7✧	0.82 (0.71-0.94)	0.73 (0.65-0.83)	0.64 (0.54-0.76)	0.74 (0.61-0.90)
CCRT	3/2❁	0.76 (0.62-0.93)	0.83 (0.68-1.02)	0.75 (0.58-0.97)	0.80 (0.57-1.11)
AC	3	0.99 (0.78-1.25)	0.86 (0.68-1.07)	0.80 (0.60-1.07)	0.85 (0.61-1.18)
CCRT plus AC	4	0.67 (0.54-0.83)	0.64 (0.52-0.79)	0.68 (0.52-0.89)	0.55 (0.38-0.79)
**Years of survival rate**					
≤3					
IC	7/4▪	0.82 (0.66-1.00)	0.74 (0.63-0.87)	0.60 (0.48-0.75)	0.73 (0.50-1.07)
CCRT	1§	0.65 (0.37-1.14)	0.75 (0.49-1.17)	0.53 (0.30-0.93)	0.95 (0.50-1.82)
AC	2	1.24 (0.82-1.86)	0.85 (0.55-1.30)	1.23 (0.77-1.97)	0.76 (0.52-1.13)
CCRT plus AC	2	0.50 (0.38-0.66)	0.47 (0.36-0.63)	0.41 (0.28-0.60)	0.52 (0.33-0.81)
≥5					
IC	6	0.85 (0.74-0.98)	0.72 (0.63-0.82)	0.72 (0.61-0.86)	0.74 (0.63-0.86)
CCRT	5/4&	0.72 (0.60-0.86)	0.77 (0.65-0.92)	0.72 (0.58-0.90)	0.67 (0.53-0.85)
AC	2	0.89 (0.67-1.16)	0.87 (0.68-1.11)	0.69 (0.50-0.97)	0.87 (0.52-1.43)
CCRT plus AC	6	0.70 (0.58-0.86)	0.70 (0.58-0.84)	0.73 (0.58-0.93)	0.66 (0.49-0.89)
**WHO histological type**					
with WHO type I					
IC	5/4	0.87 (0.75-1.02)	0.73 (0.63-0.84)	0.73 (0.61-0.89)	0.73 (0.62-0.86)

© 2019 Zhang B et al. *JAMA Network Open*.

CCRT	3/4✓	0.65 (0.51-0.83)	0.69 (0.55-0.86)	0.62 (0.46-0.82)	0.60 (0.46-0.80)
AC	3	1.08 (0.81-1.43)	0.85 (0.64-1.13)	0.86 (0.60-1.23)	0.76 (0.53-1.10)
CCRT plus AC	2#	0.59 (0.45-0.79)	0.62 (0.46-0.83)	0.44 (0.28-0.71)	0.65 (0.44-0.97)
without WHO type I					
IC	8/6	0.79 (0.65-0.95)	0.73 (0.63-0.84)	0.61 (0.50-0.75)	0.77 (0.58-1.02)
CCRT	3/2✓	0.79 (0.62-1.00)	0.90 (0.70-1.16)	0.78 (0.58-1.04)	0.91 (0.63-1.33)
AC	1	0.83 (0.57-1.21)	0.88 (0.64-1.21)	0.81 (0.53-1.24)	0.91 (0.51-1.62)
CCRT plus AC	4a	0.64 (0.53-0.78)	0.62 (0.52-0.75)	0.63 (0.50-0.79)	0.59 (0.43-0.81)
**Tumor stage**					
with stage II					
IC	5	0.93 (0.73-1.19)	0.91 (0.75-1.10)	0.68 (0.51-0.90)	0.79 (0.61-1.01)
CCRT	3	0.88 (0.65-0.99)	0.83 (0.68-1.02)	0.82 (0.61-1.09)	0.85 (0.59-1.24)
AC	1	1.37 (0.86-2.16)	0.80 (0.46-1.39)	1.18 (0.69-2.03)	0.84 (0.55-1.28)
CCRT plus AC	2	0.67 (0.45-1.00)	0.72 (0.52-0.99)	0.65 (0.44-0.95)	0.93 (0.61-1.44)
without stage II					
IC	8/7❄	0.81 (0.71-0.93)	0.67 (0.59-0.75)	0.67 (0.57-0.79)	0.72 (0.60-0.85)
CCRT	3/2⌖	0.58 (0.43-0.77)	0.65 (0.48-0.87)	0.58 (0.43-0.78)	0.63 (0.48-0.83)
AC	3	0.88 (0.68-1.14)	0.87 (0.70-1.10)	0.75 (0.54-1.02)	0.77 (0.49-1.20)
CCRT plus AC	5a	0.62 (0.52-0.74)	0.60 (0.50-0.71)	0.61 (0.49-0.77)	0.50 (0.37-0.67)
**Median follow-up time (months)**					
>60					
IC	3	0.81 (0.68-0.96)	0.74 (0.62-0.87)	0.79 (0.63-0.97)	0.71 (0.57-0.88)
CCRT	4/3◆	0.75 (0.62-0.90)	0.81 (0.67-0.97)	0.76 (0.61-0.95)	0.68 (0.53-0.86)
AC	1	0.83 (0.57-1.21)	0.88 (0.64-1.21)	0.81 (0.53-1.24)	0.91 (0.51-1.62)
CCRT plus AC	4$	0.70 (0.57-0.86)	0.69 (0.56-0.83)	0.73 (0.57-0.94)	0.67 (0.49-0.92)
≤60					
IC	10	0.87 (0.74-1.02)	0.72 (0.64-0.82)	0.60 (0.50-0.73)	0.76 (0.63-0.93)
CCRT	2§	0.52 (0.32-0.85)	0.65 (0.45-0.93)	0.45 (0.27-0.73)	0.81 (0.48-1.38)
AC	3	1.08 (0.81-1.43)	0.85 (0.64-1.13)	0.86 (0.60-1.23)	0.76 (0.53-1.10)
CCRT plus AC	3	0.52 (0.40-0.67)	0.52 (0.40-0.68)	0.46 (0.32-0.64)	0.52 (0.34-0.79)
**Study bias**					
Low-bias					
IC	9/8✡	0.80 (0.69-0.93)	0.66 (0.58-0.75)	0.62 (0.52-0.73)	0.71 (0.60-0.84)
CCRT	2§	0.52 (0.32-0.85)	0.65 (0.45-0.93)	0.45 (0.27-0.73)	0.81 (0.48-1.38)
AC	3	0.88 (0.68-1.14)	0.87 (0.70-1.10)	0.75 (0.54-1.02)	0.77 (0.49-1.20)
CCRT plus AC	5	0.68 (0.56-0.83)	0.66 (0.55-0.79)	0.67 (0.54-0.84)	0.66 (0.49-0.88)
moderate or high bias					

© 2019 Zhang B et al. *JAMA Network Open*.

IC	4	0.90 (0.75-1.08)	0.87 (0.73-1.04)	0.81 (0.63-1.02)	0.82 (0.62-1.08)
CCRT	4/3◆	0.75 (0.62-0.90)	0.81 (0.67-0.97)	0.76 (0.61-0.95)	0.68 (0.53-0.86)
AC	1	1.37 (0.86-2.16)	0.80 (0.46-1.39)	1.18 (0.69-2.03)	0.84 (0.55-1.28)
CCRT plus AC	2a	0.53 (0.40-0.70)	0.53 (0.39-0.72)	0.46 (0.30-0.71)	0.51 (0.31-0.82)

Note: IC, induction chemotherapy; AC, adjuvant chemotherapy; CCRT, concomitant chemotherapy; OS, overall survival; PFS, progression-free survival; DMFS, distance metastasis free survival; LRFS, locoregional recurrence free survival.

$ one trial had two comparisons (NPC-9902-AF and NPC-9902-CF).

* three single center trials for LRFS, and seven multiple center trials for LRFS.

¶ one trial had two comparisons (QMH-95-01 and QMH-95-04), three single center trials for PFS.

❖ seven trials with sample size >250 for LRFS and three trials with sample size ≤250 for LRFS.

❁ one trial had two comparisons (QMH-95-01 and QMH-95-04) and two trials for PFS.

✧ three trials with years of study entry >5 for LRFS and seven trials with years of study entry ≤5 for LRFS.

▪ four trials for LRFS.

§ one trial had two comparisons (QMH-95-01 and QMH-95-04).

& four trials for PFS.

✓ one trial had two comparisons (NPC-9902-AF and NPC-9902-CF), four trials with WHO histological type I for PFS and two trials without WHO histological type I for PFS.

WHO histological type of one trial (NPC-0502) was unavailable.

❄ seven trials for LRFS.

⌖ two trial for PFS.

◆ three trials for PFS.

✡ eight trials for LRFS.

© 2019 Zhang B et al. *JAMA Network Open*.

eFigure 9. Funnel plots of overall survival (a), progression-free survival (b), distance metastasis free survival (c), and locoregional recurrence free survival (d).

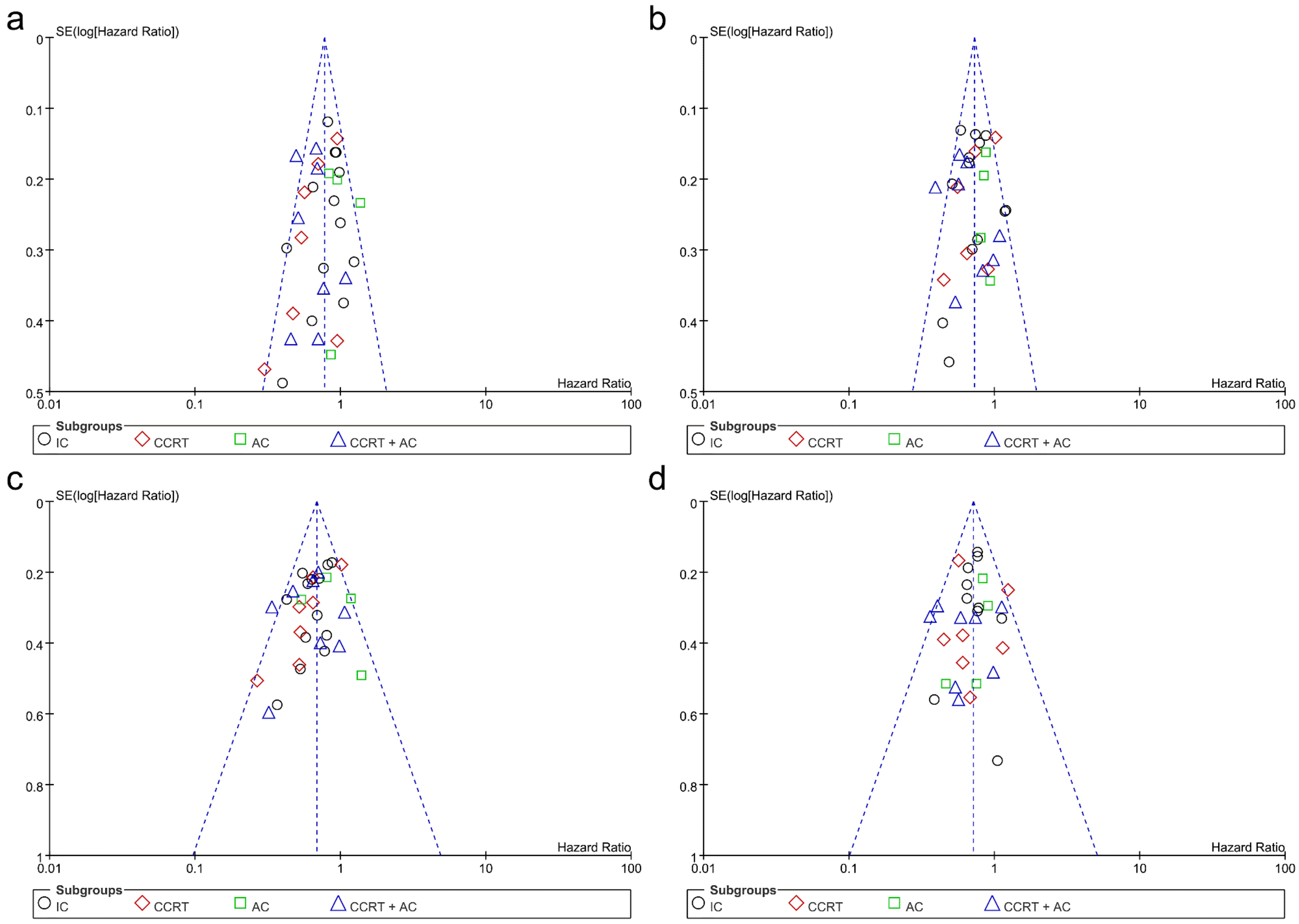

Abbreviations: OS = overall survival; PFS = progression-free survival; DMFS = distance metastasis free survival; LRFS = locoregional recurrence free survival; IC = induction chemotherapy; CCRT = concurrent chemoradiotherapy; AC = adjuvant chemotherapy.

© 2019 Zhang B et al. *JAMA Network Open*.

eTable 5. Severe (grades 3-5) toxicities related to chemoradiotherapy regimens.

Of the 28 RCTs, 21 were meta analyzed. The severe toxicities of AC regimen could not be analyzed because of no available or enough data.

Severe toxicity	Number of trials	Odds Ratio (95% CI)	Efficacy p value	Heterogeneity	
				I^2	p value
**IC plus RT vs. RT**					
Hematologic					
Anemia	-	-	-	-	-
Leukopenia	-	-	-	-	-
Thrombocytopenia	-	-	-	-	-
Febrile neutropenia	-	-	-	-	-
Phlebitis	-	-	-	-	-
Digestive system	-	-	-	-	-
Mucositis	2	1.029 (0.706-1.499)	0.883	0	0.445
Stomatitis	-	-	-	-	-
Nausea	-	-	-	-	-
Vomiting	-	-	-	-	-
Nephrotoxicity	-	-	-	-	-
Infection	-	-	-	-	-
Skin	2	0.767 (0.356- 1.651)	0.497	45.5%	0.176
Cardiac	-	-	-	-	-
Hair loss	-	-	-	-	-
Pulmonary	-	-	-	-	-
**IC plus CCRT vs. CCRT**					
Hematologic					
Anemia	4	1.867 (0.512- 6.814)	0.344	77.0%	0.005
Leukopenia	6	1.794 (1.221-2.637)	0.003	70.7%	0.004
Neutropenic	6	2.239 (1.070-5.066)	0.033	84.8%	<0.001
Febrile neutropenia	3	3.993 (1.125-14.173)	0.032	3.0%	0.357
Neutropenic infection	3	1.456 (0.192-11.050)	0.716	0%	0.526
Thrombocytopenia	7	8.488 (2.979 -24.185)	<0.001	59.1%	0.023
Hemoglobin	2	5.341 (0.883-32.319)	0.068	60.9%	0.110
Digestive system					
Diarrhea	2	1.443 (0.428-4.864)	0.554	0%	0.847
Dry mouth	4	1.171 (0.631-2.174)	0.617	41%	0.166
Hoarseness	1	-	-	-	-
Odynophagia	1	-	-	-	-
Dysphagia	4	0.759 (0.364-1.582)	0.462	36.2%	0.195
Oesophagitis	0	-	-	-	-
Mucositis	5	0.945 (0.717-1.245)	0.688	41.1%	0.147

© 2019 Zhang B et al. *JAMA Network Open*.

Stomatitis	0	-	-	-	-
Esophagitis	1	-	-	-	-
Pharynx	0	-	-	-	-
Anorexia	0	-	-	-	-
Nausea	5	1.510 (1.129-2.019)	0.005	0%	0.787
Vomiting	5	0.983 (0.565-1.710)	0.951	70.2%	0.001
Constipation	1	-	-	-	-
Bone marrow suppression	0	-	-	-	-
Hepatotoxicity					
ALT	2	1.842 (0.498-6.821)	0.360	0%	0.733
AST	5	1.504 (0.354-6.389)	0.581	36.6%	0.177
Bilirubin	1	-	-	-	-
Fatigue	2	4.605 (1.509 -14.057)	0.007	0%	0.400
Asthenia	0	-	-	-	-
Nephrotoxicity					
Dehydration	1	-	-	-	-
Electrolytes	3	1.474 (0.368-5.909)	0.584	31.6%	0.232
Creatinine	1	-	-	-	-
Epistaxis	1	-	-	-	-
Eye damage					
Conjunctivitis/keratitis	3	0.527 (0.070-3.962)	0.533	0%	0.814
Ototoxicity	4	0.955 (0.587-1.533)	0.851	0%	0.939
Infection	2	0.685 (0.114-4.117)	0.679	0%	0.660
Fever	1	-	-	-	-
Neurotoxicity					
Temporal lobe necrosis	2	0.330 (0.034-3.176)	0.337	0%	0.519
Cranial neuropathy	2	0.827 (0.250-2.729)	0.755	0%	0.820
Peripheral neuropathy	2	5.008 (0.583-43.016)	0.142	0%	0.700
Hemorrhage	3	0.914 (0.094-8.872)	0.938	0%	0.994
Musculoskeletal					
Bone necrosis	2	0.425 (0.063-2.886)	0.381	0%	0.434
Allergenic reaction	2	3.004 (0.311-28.974)	0.341	0%	0.521
Skin					
Dermatitis	4	0.595 (0.332-1.066)	0.081	35.1%	0.201
Rash	1	-	-	-	-
Desquamation	1	-	-	-	-
Soft tissue damage					
neck	2	0.425 (0.062-2.889)	0.381	0%	0.632
Trismus	2	0.996 (0.062-15.968)	0.998	0%	0.998
Transfusion	1	-	-	-	-
Irregular menses	1	-	-	-	-
Lymphoedema (submental)	1	-	-	-	-

© 2019 Zhang B et al. *JAMA Network Open*.

Pain	1	-	-	-	-
Hair loss	1	-	-	--	
Pulmonary hypoxia	1	-	-	-	-
Second cancer (primary site)	1	-	-	-	-
Weight loss	3	1.043 (0.565-1.924)	0.893	0%	0.957
**CCRT vs. RT**					
Hematologic					
Anemia	1	-	-	-	-
Leukopenia	3	14.941 (2.877-77.581)	0.001	0%	0.485
Neutropenic	0	-	-	-	-
Thrombocytopenia	4	2.190 (0.488-9.839)	0.306	0%	0.974
Hemoglobin	1	-	-	-	-
Digestive system					
Diarrhea	1	-	-	-	-
Dry mouth	1	-	-	-	-
Mucosal damage	1	-	-	-	-
Mucositis	4	2.555 (1.145-5.701)	0.022	83.6%	0.0004
Nausea/vomiting	3	9.302 (2.155-40.154)	0.003	0%	0.425
Dysphagia	1	-	-	-	-
Hepatotoxicity	1	-	-	-	-
Nephrotoxicity	1	-	-	-	-
Ototoxicity	2	1.330 (0.656-2.697)	0.429	0%	0.651
Skin fibrosis	4	2.104 (1.110-3.989)	0.023	65.1%	0.035
Neurotoxicity	1	-	-	-	-
soft tissue damage	1	-	-	-	-
Trismus	1	-	-	-	-
Weight loss	2	0.662 (0.232-1.890)	0.441	0%	0.465
**CCRT plus AC vs. RT**					
Hematologic					
Anemia	3	7.318 (1.618- 33.098)	0.010	0%	0.560
Leukopenia	4	41.95 (13.07-134.64)	<0.001	0%	0.710
Neutropenic	1	-	-	-	-
Febrile neutropenia	1	-	-	-	-
neutropenic infection	1	-	-	-	-
Thrombocytopenia	5	3.250 (1.048-10.080)	0.041	0%	0.463
Granulocytopenia	1	-	-	-	-
Digestive system					
Anorexia	1	-	-	-	-
Diarrhea	2	1.049 (0.107- 10.285)	0.041	0%	0.882
Dry mouth	0	-	-	-	-
Mucositis	3	1.923 (1.271-2.909)	0.002	0%	0.935

© 2019 Zhang B et al. *JAMA Network Open*.

Stomatitis	3	6.005 (0.670-53.795)	0.109	68.4%	0.042
Hepatitis	2	2.081 (0.191-22.704)	0.548	0%	0.568
Taste alternation	0	-	-	-	-
Nausea	1	-	-	-	-
Vomiting	5	15.137 5.451-42.034)	<0.001	0%	0.641
Dysphagia	4	1.529 (0.349-6.693)	0.573	18.3%	0.299
Constipation	2	1.908 (0.166-21.981)	0.604	0%	0.770
Metabolic disturbance	2	2.081 (0.191-22.704)	0.548	0%	0.568
Endocrine dysfunction	2	1.049 (0.206-5.352)	0.954	0%	0.832
Syncope	2	2.081 (0.191-22.704)	0.548	0%	0.568
Nephrotoxicity	2	3.984 (0.432-36.734)	0.223	0%	0.940
Eye damage	4	1.498 (0.422-5.320)	0.532	0%	0.843
Ototoxicity	5	1.725 (1.261-2.359)	0.001	0%	0.835
Infection					
Non-neutropenic	3	2.458 (0.479-12.610)	0.281	0%	0.699
Skin					
Desquamation	5	1.327 (0.795 -2.214)	0.279	0%	0.451
Erythema	4	1.433 (0.838-2.450)	0.189	0%	0.427
Neurotoxicity					
Symptomatic brain damage	1	-	-	-	-
Temporal lobe necrosis	4	0.845 (0.380-1.881)	0.680	0%	0.626
Brainstem damage	1	-	-	-	-
Spinal damage	1	-	-	--	-
Cranial neuropathy	4	1.259 (0.428-3.702)	0.676	54.5%	0.086
Peripheral neuropathy	4	2.843 (0.889-9.091)	0.078	0%	0.418
Brachial plexopathy	3	0.435 (0.063-3.017)	0.400	0%	0.938
Bone necrosis	2	1.328 (0.141-12.533)	0.804	27.3%	0.241
Vascular damage	3	1.051 (0.318-3.467)	0.935	0%	0.516
soft tissue damage					
Nasopharynx	3	0.964 (0.495-1.879)	0.914	0%	0.819
Neck	3	1.703 (0.238-12.198)	0.596	60.8%	0.078
Radiation-induced malignancy					
Weight loss	1	-	-	-	-
Others	0	--	-	-	-

© 2019 Zhang B et al. *JAMA Network Open*.
